# Semen quality in men with cancer: comparison with a healthy Swiss reference population

**DOI:** 10.1530/RAF-26-0110

**Published:** 2026-10-13

**Authors:** Myriam Vogt, Rita Rahban, Benedetta Campana, Gyongyver Teglas, Alfred Senn, Cyril Jaksic, Serge Nef, Alexandra Kohl Schwartz, Marcel Steinmann

**Affiliations:** ^1^ Lucerne Cantonal Hospital, University Teaching and Research Hospital of The University of Lucerne, Division of Gynaecological Endocrinology and Reproductive Medicine, Lucerne, Switzerland; ^2^ University of Geneva, Department of Genetic Medicine and Development, Geneva, Switzerland; ^3^ Swiss Centre for Applied Human Toxicology (SCAHT), Basel, Switzerland; ^4^ Lucerne Cantonal Hospital, University Teaching and Research Hospital of The University of Lucerne, Division of Medical Oncology, Lucerne, Switzerland; ^5^ Geneva University Hospitals, Clinical Research Centre, Geneva, Switzerland; ^6^ University Hospital Bern, Obstetrics and Gynaecology, University of Bern, Bern, Switzerland

**Keywords:** semen quality, cancer, tumour, male fertility, chemotherapy, gonadotoxicity, fertility preservation, cryopreservation

## Abstract

**Abstract:**

This study aimed to investigate the impact of cancer and its treatment on semen quality and to examine the differences according to cancer type and stage. This monocentric retrospective cohort study included men aged 12–50 years who underwent semen analysis for fertility preservation prior to cancer treatment between 2005 and 2024 at a public hospital in Central Switzerland. Semen parameters of 373 cancer patients were compared with those of a healthy Swiss reference population (2,871 military recruits) with adjustment for potential confounders. Subgroup analyses were performed by cancer type and stage, while paired analyses evaluated changes in semen quality before and after treatment. Before treatment, cancer patients had lower sperm concentration, total sperm count and total motile sperm count compared with the reference population. After adjustment, these differences remained significant only in men with testicular cancer. Following treatment, the proportion of men with azoospermia increased across all cancer types. While partial recovery was observed in patients with testicular cancer, patients with haematological and other solid cancers demonstrated a clear deterioration in semen quality, consistent with the higher gonadotoxicity of their treatments. In conclusion, semen quality may already be compromised in men with cancer prior to treatment, especially in testicular cancer. The extent of treatment-related impairment varies by cancer type and treatment. These findings highlight the importance of individualised and timely fertility counselling, sperm cryopreservation prior to treatment and the need for structured long-term follow-up of reproductive health in cancer survivors.

**Lay summary:**

Cancer and its treatment can reduce fertility in men of reproductive age and can affect the ability to have children. It is still unclear how much of this effect is caused by the disease itself and how much by the treatment. In this study, we compared semen quality in men with cancer before and after treatment with that of healthy Swiss men. We found that some patients, especially those with testicular cancer, already had lower semen quality before starting the treatment. After treatment, semen quality often further decreased and more men had no sperm in their ejaculate. This was particularly seen in patients who received treatments known to strongly affect fertility. These findings show that both cancer and cancer treatment can impair male fertility. This highlights the importance of early counselling and offering sperm freezing before treatment, as well as providing long-term reproductive follow-up after cancer care.

## Introduction

An increasing number of men of reproductive age are being diagnosed with cancer (Miller *et al.* 2020). In Switzerland, the incidence of cancer among men up to 54 years old increased by approximately 51% between 1981 and 2020 (National Agency for Cancer Registration (NACR) 2023). In 2022, the most common cancer among young European men (15–39 years old) was testicular cancer (26.6%). This was followed by melanoma (10.1%), Hodgkin lymphoma (7.7%), brain and central nervous system (CNS) cancer (7.2%) and non-Hodgkin lymphoma (6.9%) (International Agency for Research on Cancer (IARC) 2026).

Improved cancer treatments have led to higher survival rates (Mertens *et al.* 2008, Trama *et al.* 2012, Hoshi *et al.* 2014, Miller *et al.* 2020) but may also impair male fertility (Schover *et al.* 2002, Tournaye *et al.* 2014, Lambertini *et al.* 2016). Cancer treatments, including surgery, chemotherapy and radiotherapy, are well known to exert gonadotoxic effects and can substantially reduce semen quality or cause azoospermia depending on treatment type and cumulative dose (Petersen *et al.* 1999, Vakalopoulos *et al.* 2015, Halpern *et al.* 2020, Parekh *et al.* 2020). The impact of new cancer treatments such as targeted therapies and immune checkpoint inhibitors on male fertility remains poorly understood, with limited and conflicting clinical data (Chang *et al.* 2017, Traila *et al.* 2018, Garutti *et al.* 2021, Rambhatla *et al.* 2021, Hassel & Berking 2023, Himpe *et al.* 2023). Combined oncological treatments are associated with higher gonadotoxicity than single modalities (Barrass *et al.* 2004).

While the gonadotoxic effects of conventional cancer treatment are well established, increasing evidence suggests that the malignancy itself may impair semen quality even before treatment (Chung *et al.* 2004, Trottmann *et al.* 2007, Kawai & Nishiyama 2019, Liu *et al.* 2021, Pening *et al.* 2022, Shrem *et al.* 2022, Peluso *et al.* 2023). Reduced semen quality has been reported particularly in men with testicular and haematological cancer, with lower cryopreservation success rates observed in the latter (Yumura *et al.* 2018, Peluso *et al.* 2023). The underlying mechanisms remain incompletely understood but may involve disruption of the hypothalamic–pituitary–gonadal axis, systemic inflammation, cachexia, fever and psychological distress (Halpern *et al.* 2020, Brannigan *et al.* 2021). The impact appears to vary by cancer type, with testicular tumours associated with local damage and hormonal alterations, and haematological malignancies reflecting systemic disease (Agarwal & Allamaneni 2005, Williams *et al.* 2009, Vakalopoulos *et al.* 2015, Yumura *et al.* 2018, Pening *et al.* 2022, Peluso *et al.* 2023). In addition, lifestyle and medical factors, including obesity, smoking, alcohol use and prior conditions, such as cryptorchidism or varicocele, may further affect semen quality (Verón *et al.* 2018, Rahban *et al.* 2019).

Given the potential impact of both cancer and its treatment on male fertility, the ability to have biological children remains a major concern for men of reproductive age (Vakalopoulos *et al.* 2015, Lehmann *et al.* 2017). Infertility can cause significant psychological distress in young cancer patients particularly when biological parenthood may no longer be possible (Schover 2009).

In this monocentric retrospective cohort study, we investigated the impact of cancer and its treatment on male fertility in patients from a public hospital in Central Switzerland. Semen quality parameters were compared between cancer patients and a healthy Swiss reference population. In addition, the influence of cancer type and stage, comorbidities and lifestyle factors on semen quality was assessed. The findings aim to improve understanding the effects of cancer and its treatment on male reproductive health, and to support fertility counselling and preservation strategies.

## Materials and methods

### Study population

#### Cancer patients

Eligible participants were male cancer patients aged 12–50 years who were referred to the Lucerne University Teaching and Research Hospital (hereafter referred to as Lucerne) in Switzerland between January 2005 and December 2024 for sperm cryopreservation prior to oncological treatment. The upper age limit of 50 years was chosen to minimise potential confounding by age-related changes in semen quality, which become increasingly relevant with advancing paternal age (Paoli *et al.* 2019). In addition, as fertility preservation in Switzerland is reimbursed only up to the age of 40 years, relatively few patients above this age were expected in our cohort. Exclusion criteria were prior or ongoing systemic cancer treatment and documented refusal of consent. A total of 373 male cancer patients aged 13–50 years met the inclusion criteria. No *a priori* sample size calculation was performed, as all eligible patients available during the predefined study period were included. The study selection process is shown in Fig. 1. During the study period, 549 semen samples of cancer patients were analysed for cryopreservation at the laboratory of reproductive medicine in Lucerne. As some patients provided multiple semen samples, only the first sample per patient was included, resulting in the removal of 82 duplicates and 467 individual cancer patients. Of these, 94 were excluded due to age > 50 years (*n* = 11), prior systemic cancer treatment (*n* = 32), written refusal of consent (*n* = 17) or insufficient patient data (*n* = 34), leaving 373 patients for analysis. Patients were categorised into three groups according to malignancy type: testicular cancer (TC), haematological cancer (HC) and other solid cancers (OSCs). Semen analysis results and medical records were retrieved from hospital information systems and paper files primarily used before 2013. Collected variables included demographics, medical history, cancer diagnosis and staging, as well as semen parameters at the time of cryopreservation prior to cancer treatment and at follow-up after treatment. Data on fertility outcomes, specifically live births, were retrieved from the Swiss National Civil Registry. All data were pseudonymised prior to analysis to ensure patient confidentiality.

**Figure 1:**
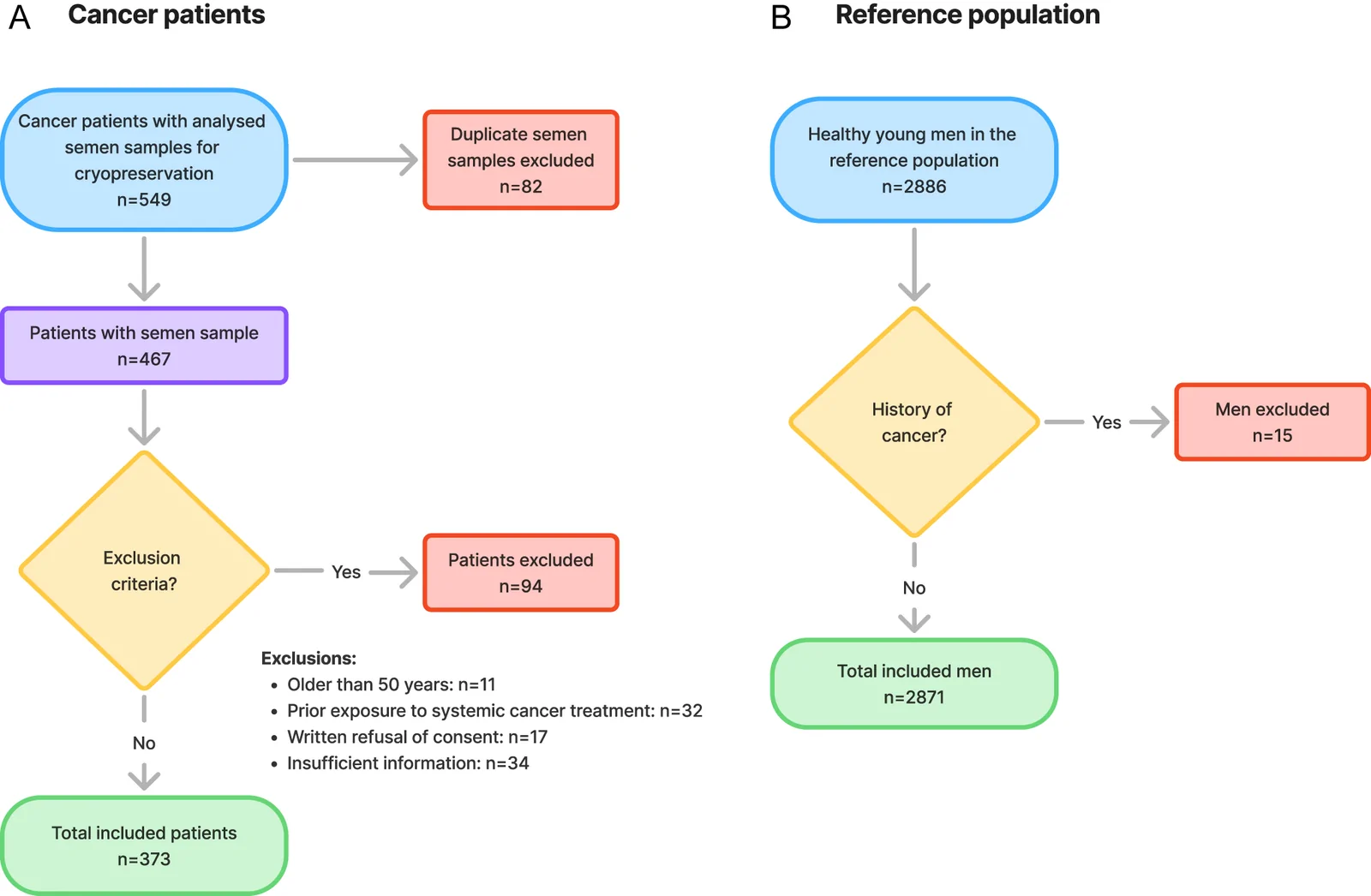
Flowchart of study population selection for cancer patients and the reference population. Flow diagram illustrating the inclusion and exclusion process for (A) cancer patients and (B) the reference population. Among 549 semen samples analysed for cryopreservation between 2005 and 2024 in Lucerne, 82 duplicates were removed and 467 individual patients remained. Following application of exclusion criteria, 94 patients were excluded, resulting in a final cohort of 373 cancer patients. For the reference population, 2,886 men were initially included. After exclusion of individuals with a history of cancer (*n* = 15), 2,871 men remained for analysis.

#### Reference population

The reference dataset was obtained from a Swiss nationwide cross-sectional study on semen quality, as previously described (Rahban *et al.* 2019, 2023). Nearly all Swiss men aged 18–22 years (approximately 97%) attend a mandatory military recruitment assessment and were invited to participate in the study between 2005 and 2018. Recruitment was conducted across six army recruitment centres in Switzerland (Lausanne, Windisch, Monteceneri, Rüti, Sumiswald and Mels). Participants received study information, a consent form and standardised questionnaires addressing personal health, lifestyle and parental factors during the preconception period. In addition, semen analyses and standardised urological examinations were performed. Of 92,274 men contacted, 4,837 (5.3%) returned completed questionnaires and 2,886 (3.0%) provided biological samples. No specific inclusion or exclusion criteria were applied at recruitment, allowing inclusion of men with diverse fertility profiles and reflecting a broader population rather than restricting the reference group to proven fertile men, as in the populations used to establish the World Health Organization (WHO) semen reference parameters. For the present analysis, individuals with prior or current cancer (*n* = 15) were excluded, resulting in a final study population of 2,871 men (Fig. 1).

### Sample collection

In cancer patients, semen samples were obtained by masturbation following a recommended sexual abstinence period of 2–5 days and were preferentially collected on-site in a private room within the andrology laboratory. In a minority of cases, samples were collected off-site (at home or in the oncology ward) and transported using sterile containers (Vitrolife Sperm Collection Cup, Sweden; Urine container, SARSTEDT, Germany). The exact proportion of off-site collections could not be reliably determined. In two cases where orthograde ejaculation was not possible, post-masturbation urine samples were analysed for the presence of sperm; both showed azoospermia and were, therefore, included in the analysis. Testicular sperm extraction (TESE) was performed in 11 cases due to azoospermia or very low sperm concentration precluding cryopreservation. However, these data were not included in the main analysis, as sperm concentration and motility cannot be reliably compared with ejaculated samples. Prior to initiation of any cancer treatment, blood samples for hormonal analysis were obtained in 39 patients, and total testosterone and follicle-stimulating hormone (FSH) were measured. Hormonal measurements were available predominantly for TC patients treated in more recent years as part of routine clinical care, without predefined selection criteria. Data for HC and OSCs were too limited for meaningful subgroup analyses; therefore, hormonal analyses were restricted to TC patients.

For the reference population, semen samples were collected by masturbation following a recommended sexual abstinence period of at least 2 days. The semen sample was collected in a sterile, non-toxic 15 mL container equipped with an integrated funnel (Spermo-Sampler, Verridial, Blonay VD, Switzerland).

### Semen analysis

Semen samples from cancer patients were analysed at the laboratory of reproductive medicine in Lucerne using standardised procedures and conventional manual microscopy in accordance with the WHO guidelines for semen analysis (World Health Organization 1999, 2010). The 2021 WHO guideline had not yet been implemented in the laboratory during the study period. Recorded parameters included semen volume, sperm concentration, total sperm count (TSC), sperm motility and sperm morphology. Derived measures such as the total motile sperm count (TMSC) were calculated accordingly, and abstinence time was recorded. Some parameters (e.g. semen pH, progressive motility) were not consistently available, particularly in earlier years. Sperm morphology was not systematically assessed at the time of cryopreservation and was, therefore, excluded from the analysis. In the reference population, semen samples were analysed using computer-assisted sperm analysis (CASA, Sperm Class Analyzer, SCA, Microptic, Barcelona, Spain). Samples were diluted with a HEPES-buffered medium containing 0.4% HSA (IVF basics, Gynotec B.V, GC Malden, the Netherlands) when needed. Aliquots of 5 μL were loaded into a 20 μm counting chamber (Leja Products, GN Nieuw-Vennep, the Netherlands) and examined on a thermostatically controlled microscope stage at 37°C. Observations were made with a 10× phase contrast objective at a final 100× magnification. For each sample, a minimum of 500 sperm tracks were captured at a rate of 25 images per second. Identical CASA systems were used in all study locations.

### Gonadotoxicity

A classification matrix was developed to assess the gonadotoxic effects of various cancer therapies based on current literature, clinical guidelines and the clinical expertise of the Department of Medical Oncology of Lucerne (Supplementary Table 1 (see section on Supplementary materials given at the end of the article)) (Dohle 2010, Chang *et al.* 2017, Dittrich *et al.* 2018, Lambertini *et al.* 2020, Von Wolff & Nawroth 2020, Brannigan *et al*. 2021, Rambhatla *et al.* 2021, Mauz-Körholz *et al.* 2022 Pagano *et al.* 2022, Himpe *et al.* 2023, Xue *et al.* 2023). This framework was used to categorise the gonadotoxicity levels of therapies received by cancer patients and to standardise data analysis and interpretation. Gonadotoxicity was classified into three categories according to the estimated risk of permanent azoospermia: low (<20%), moderate (20–80%) and high (>80%), with an additional ‘unknown’ category when evidence was insufficient or inconclusive. This categorisation was based on previously published clinical guidelines and risk classifications for treatment-related gonadotoxicity (Muñoz *et al.* 2016, Lambertini *et al.* 2020).

### Statistical analysis

Continuous variables are reported descriptively as both means with standard deviations (SD) and medians with interquartile ranges (IQR; 25th–75th percentile), where applicable. For paired pre- and post-treatment analyses, continuous variables are presented as means with SD together with mean differences and 95% confidence intervals. Categorical variables are reported as counts and percentages. Simple comparisons between all cancer patients and the reference population were performed using Welch’s *t*-tests for continuous outcomes and chi-squared tests (or Fisher’s exact tests when at least one expected cell count was lower than 5) for categorical outcomes. Given the large sample sizes in the between-group comparison, formal normality testing was not used as a prerequisite for Welch’s *t*-tests, which were considered robust to deviations from normality based on the sampling distribution of the mean difference (Curran-Everett 2017, Tsagris *et al.* 2020). To assess differences in semen quality parameters between groups, linear regression analyses were performed with group as the predictor of interest and the respective semen parameter as the outcome, adjusting for age, cryptorchidism, endocrine comorbidities, smoking status, alcohol consumption, abstinence duration and body mass index (BMI). These covariates were selected *a priori* based on their established or clinically plausible associations with semen quality. Both unadjusted and adjusted coefficients are reported with their 95% confidence intervals (CI). To test differences in semen quality parameters before and after treatment in cancer patients, paired *t*-tests were computed for continuous outcomes, while for change in proportions (categorical outcomes) the bias-corrected and accelerated (BCa) bootstrap was used with the *P*-value computed using the inversion of the confidence interval method. Analyses were performed using available data for each respective outcome and covariate; no imputation of missing data was performed. For all analyses, a bilateral *P*-value <0.05 was considered statistically significant. Analyses were conducted using R (v. 4.2.2).

### Ethical approval

The study was approved by the Swiss Association of Research Ethics Committees (BASEC ID 2024-00343) and was conducted in accordance with the principles of the Declaration of Helsinki. Patient data were used in accordance with the institutional general consent procedure; patients who had refused general consent were excluded from the analysis.

## Results

### General characteristics of the study population

The general characteristics of the study population are shown in Table 1. The included cancer patients (*n* = 373) were classified into three groups according to cancer type: testicular cancer (TC, *n* = 184, 49.3%), haematological cancer (HC, *n* = 122, 32.7%) and other solid cancers (OSCs, *n* = 67, 18%) (Fig. 2A). Detailed information on the specific cancer types within each group is provided in Supplementary Table 2.

**Table 1:** General characteristics of cancer patients according to cancer type compared with the healthy reference population. Continuous variables are presented as mean (SD) and median (IQR; 25th–75th percentile). Categorical variables are presented as *n* (%). Group comparisons were performed using the chi-square test or Fisher’s exact test when appropriate. Statistically significant *P*-values (*P* < 0.05) are shown in bold.

	Reference population		All cancers		*P*-value	Cancer type		
	*n*	Values	*n*	Values		Testicular	Haematological	Solid
General characteristics								
Total *n*	2,871			373		184 (49.3%)	122 (32.7%)	67 (18.0%)
Age (years)	2,868		373		**<0.001**			
Mean ± SD		19.7 ± 1.1		28.7 ± 7.6		29.2 ± 6.5	27.8 ± 8.2	29.1 ± 8.9
Median (IQR)		19.2 (19.0–20.0)		28.0 (23.0–34.0)		29.0 (25.0–33.0)	27.0 (22.0–34.0)	30.0 (21.0–35.0)
BMI (kg/m^2^)	2,576		283		**<0.001**			
Mean ± SD		23.0 ± 3.1		24.9 ± 4.0		25.2 ± 3.8	24.6 ± 4.4	24.7 ± 3.8
Median (IQR)		22.6 (20.9–24.5)		24.4 (22.2–27.1)		24.5 (22.9–27.2)	24.3 (21.4–27.1)	24.4 (21.6–26.9)
Medication intake*	2,871	121 (4.2%)	293	138 (47.1%)	**<0.001**	66 (46.5%)	42 (43.8%)	30 (54.5%)
Ever fathered a child^†^	2,871	2 (0.1%)	359	41 (11.4%)	**<0.001^‡^**	17 (9.7%)	17 (14.2%)	7 (10.9%)
Lifestyle factors^§^								
Cigarette smokers	2,479	504 (20.3%)	179	24 (13.4%)	**0.025**	14 (17.5%)	7 (11.5%)	3 (7.9%)
Alcohol consumers	2,781	2,244 (80.7%)	222	139 (62.6%)	**<0.001**	65 (68.4%)	42 (56.0%)	32 (61.5%)
Cannabis consumers	2,780	323 (11.6%)	221	20 (9.0%)	0.248	6 (6.5%)	8 (10.5%)	6 (11.5%)
Comorbidities^║^								
Endocrine	2,871	8 (0.3%)	315	6 (1.9%)	**0.001^‡^**	3 (2.1%)	3 (2.8%)	0 (0.0%)
Urological	2,554	614 (24.0%)	321	66 (20.6%)	0.167	43 (28.9%)	15 (13.8%)	8 (12.7%)
Others	2,870	342 (11.9%)	317	110 (34.7%)	**<0.001**	42 (29.2%)	45 (40.9%)	23 (36.5%)

BMI, body mass index.

* Any medication taken during the 3 months prior to participating in the study.

^†^ Before any cancer treatment for cancer patients.

^‡^ Fisher’s exact test.

^§^ Any reported or documented intake.

^║^ Reported or documented prior to participating in the study (listed in Supplementary Table 3).

**Figure 2:**
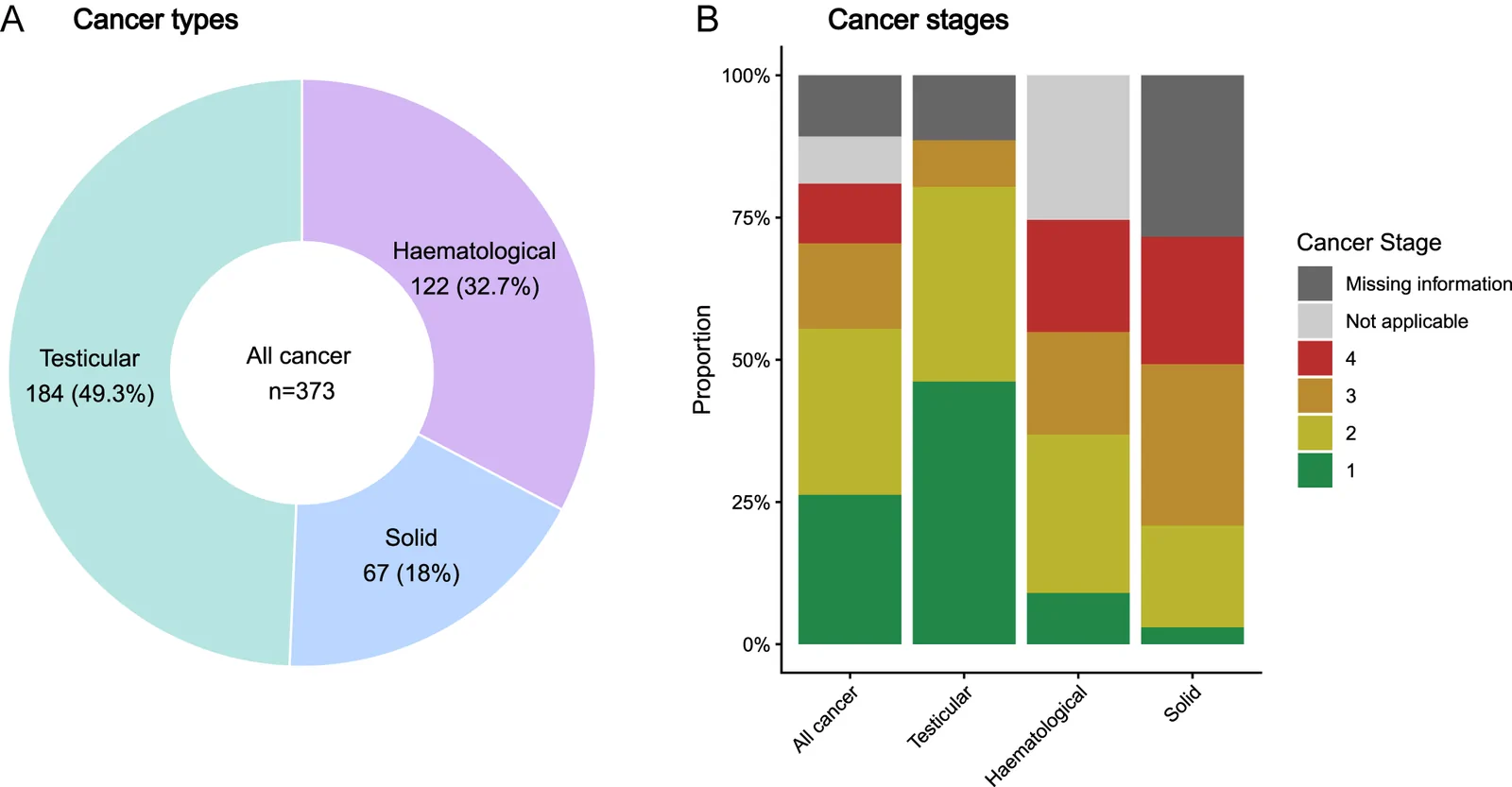
Distribution of cancer patients according to cancer type and stage. (A) Doughnut chart showing the distribution of cancer types among the study population (*n* = 373), including testicular (49.3%, *n* = 184), haematological (32.7%, *n* = 122) and other solid cancers (18.0%, *n* = 67). (B) Stacked bar chart illustrating the proportion of cancer stages (stage 1–4), as well as cases with missing or not applicable staging information, across the overall cohort and by cancer type. Proportions are presented as percentages within each group. Cancer stages were classified according to the Union Internationale Contre le Cancer (UICC) or the Ann Arbor system. The UICC classification applies to testicular cancer (stages 1–3) and other solid tumours (stages 1–4), while the Ann Arbor classification is used for Hodgkin and non-Hodgkin lymphomas (stages 1–4). Other haematological malignancies could not be categorised due to the lack of a unified classification system and were considered not applicable.

The distribution of cancer stages across different cancer types is shown in Fig. 2B. TC patients were predominantly diagnosed at early stages, with most cases classified as stage 1 or 2. By contrast, HC patients were more frequently diagnosed at more advanced stages, with stage 2 being the most common. Patients with OSCs showed the highest proportion of advanced disease, with stage 3 occurring most frequently. Overall, early-stage disease (stage 1–2) predominated in the total cancer cohort, largely reflecting the high proportion of TC.

Cancer patients were significantly older at the time of sperm cryopreservation than men in the reference population (mean age: 28.7 vs 19.7 years). Similarly, BMI was significantly higher among cancer patients, with a mean BMI of 24.9 kg/m^2^ compared with 23.0 kg/m^2^ in the reference population (Table 1). Cancer patients reported medication use more frequently within the three months prior to inclusion (47.1 vs 4.2%). Endocrine and other comorbidities were more common in the cancer cohort, while urological comorbidities did not differ significantly between groups. By contrast, alcohol and nicotine consumption were significantly more frequent in the reference population.

When stratified by cancer type, age and BMI were comparable among cancer patients. Urological comorbidities were most frequent among TC patients (28.9%), whereas they were less common in HC (13.8%) and OSC patients (12.7%). Other comorbidities (listed in Supplementary Table 3) were reported more frequently in patients with HC (40.9%) and OSCs (36.5%) than in those with TC (29.2%) (Table 1). According to the Swiss National Civil Registry, 25 cancer patients (6.7%) died during the study period; however, the cause of death was unknown.

### Pre-treatment semen parameters in cancer patients compared with the healthy reference population

Compared with the reference population, cancer patients showed reduced sperm concentration, TSC and TMSC before treatment, while semen volume and total motility were comparable (Fig. 3 and Supplementary Table 4). Impairment was most pronounced in TC, followed by HC, whereas OSC patients showed semen parameters comparable to the reference population. Across cancer groups, semen volume remained similar and total motility was largely preserved. No linear deterioration was observed with increasing cancer stage (Supplementary Table 5). Azoospermia was more frequent in cancer patients than in the reference population (3.1 vs 1.0%), particularly in TC (4.3%) and HC (2.9%), while no cases were observed in OSCs (Supplementary Table 6).

**Figure 3:**
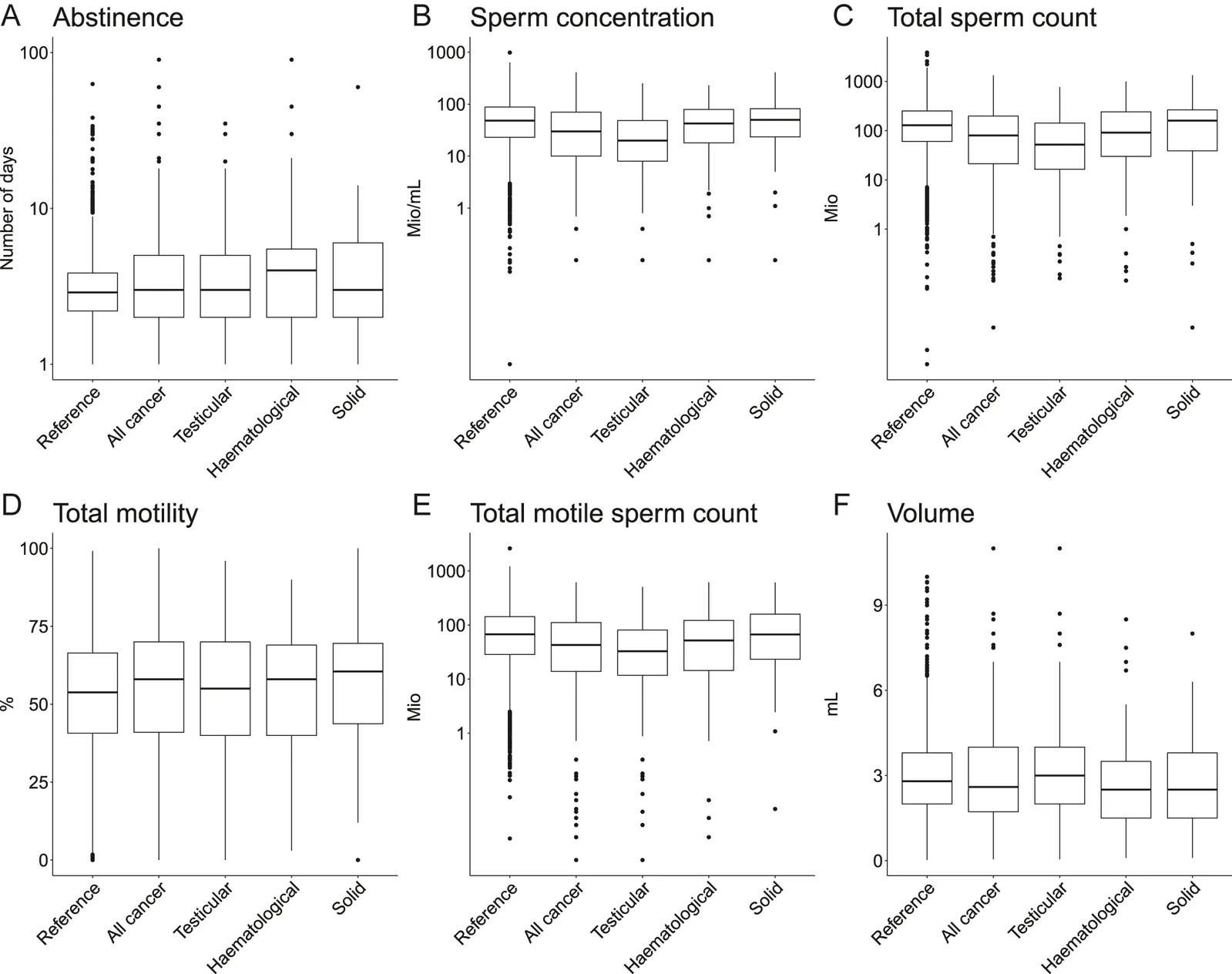
Distribution of pre-treatment semen parameters of cancer patients according to cancer type compared with the healthy reference population. Boxplots show the distribution of (A) abstinence time (days; number of days between the last ejaculation and semen sample collection); (B) sperm concentration (million/mL); (C) total sperm count (million); (D) total motility (%; sum of fast progressive, slow progressive, and non-progressive motility); (E) total motile sperm count (million; semen volume × sperm concentration × total motility) and (F) semen volume (mL) in the reference population and in cancer patients overall, as well as stratified by cancer type (testicular, haematological and other solid cancers). The central line represents the median, boxes indicate the interquartile range (IQR), whiskers extend to 1.5× IQR, and points represent outliers. The figure presents raw, unadjusted data for descriptive purposes; adjusted between-group comparisons are reported in Table 2.

After adjustment for age, history of cryptorchidism, endocrine comorbidities, nicotine and alcohol consumption, duration of abstinence and BMI, no statistically significant differences were observed between cancer patients (total cohort) and the reference population for sperm concentration, TSC, TMSC or total motility (Table 2). However, semen volume was significantly lower in cancer patients. Upon stratification by cancer type, TC patients had a significantly lower sperm concentration, TSC and TMSC compared with the reference population. By contrast, HC patients showed no significant differences in these parameters, although semen volume was significantly reduced. No significant differences were observed in patients with OSCs. Total sperm motility did not differ significantly from the reference population in any cancer group.

**Table 2:** Adjusted linear regression analyses of pre-treatment semen parameters of cancer patients according to cancer type compared with the healthy reference population. Adjustment was made for age, cryptorchidism, endocrine comorbidities, nicotine use, alcohol consumption, days of abstinence and body mass index. Statistically significant *P*-values (*P* < 0.05) are shown in bold.

	Volume (mL)			TMSC (Mio)			Concentration (Mio/mL)			Total sperm count (Mio)			Total Motility^†^(%)		
	β*	95% CI	*P*-value	β*	95% CI	*P*-value	β*	95% CI	*P*-value	β*	95% CI	*P*-value	β*	95% CI	*P*-value
Reference population	(Ref)			(Ref)			(Ref)			(Ref)			(Ref)		
All cancer patients	−0.45	−0.83 to −0.07	**0.020**	−17.11	−47.31–13.09	0.267	−8.25	−24.96–8.45	0.333	−32.61	−79.07–13.84	0.169	1.26	−3.78–6.31	0.624
Cancer type															
Testicular	0.08	−0.43–0.59	0.747	−53.34	−84.57 to −22.12	**0.001**	−38.07	−53.61 to −22.52	**<0.001**	−92.51	−143.59 to −41.42	**<0.001**	2.06	−5.01–9.13	0.568
Haematological	−0.87	−1.37 to −0.38	**0.001**	−9.50	−61.08–42.09	0.718	2.91	−21.82–27.64	0.818	−23.69	−105.48–58.10	0.570	0.30	−6.04–6.64	0.925
Solid	−0.64	−1.29–0.00	0.051	24.13	−37.16–85.41	0.440	22.58	−13.40–58.56	0.219	45.84	−36.08–127.77	0.273	1.33	−8.34–11.01	0.787

CI, confidence interval; TMSC, total motile sperm count (calculated as volume × sperm concentration × total motility).

* Computed from linear regressions and the robust Huber–White (HC3) method.

^†^ All motile sperm (a + b + c): a = fast progressive movement, b = slow progressive movement, c = non-progressive movement.

The associations between potential influencing factors and semen parameters before cancer treatment are illustrated in Fig. 4 and Supplementary Table 7. In the adjusted models, patients with cryptorchidism and endocrine comorbidities showed significantly lower sperm concentration, TSC and TMSC. Longer abstinence periods were significantly linked to higher volume, sperm concentration, TSC and TMSC, but to lower sperm motility. Age, BMI, alcohol consumption and nicotine use did not show consistent associations with semen parameters in this cohort.

**Figure 4:**
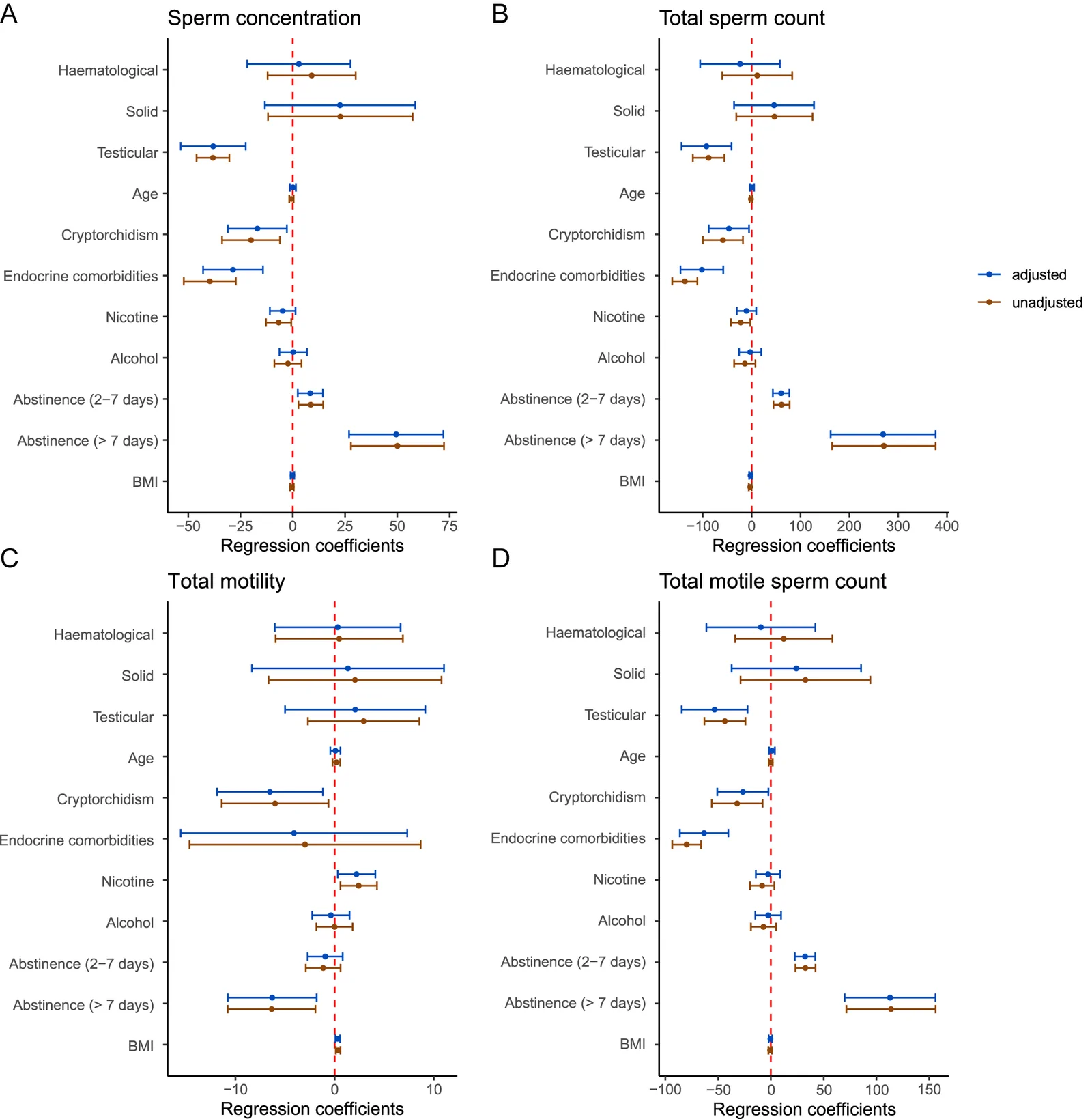
Multivariable regression analysis of factors associated with semen parameters in cancer patients. Forest plots showing regression coefficients and 95% confidence intervals for the associations between clinical and lifestyle factors and semen parameters, including (A) sperm concentration (Mio/mL), (B) total sperm count (Mio), (C) total motility (%) and (D) total motile sperm count (Mio). Results are presented as unadjusted (orange) and adjusted (blue) estimates. The dashed vertical line indicates no effect (coefficient = 0). Covariates included cancer type (testicular, haematological and other solid cancers), age, history of cryptorchidism, endocrine comorbidities, nicotine use and alcohol consumption (any reported or documented intake), abstinence time (number of days between the last ejaculation and semen sample collection) and body mass index (BMI). Reference categories were no cancer (reference population) for cancer types, absence of cryptorchidism, no endocrine comorbidities, no nicotine or alcohol use and abstinence <2 days. BMI was included as a continuous variable. Regression coefficients were estimated using linear regression with robust Huber–White (HC3) standard errors. Total motility refers to the proportion of all motile sperm (sum of fast progressive, slow progressive and non-progressive motility). Total motile sperm count was calculated as semen volume × sperm concentration × total motility.

### Subgroup analysis of testicular function in testicular cancer patients

A subgroup analysis comparing TC patients with the reference population showed that TC patients had significantly higher follicle-stimulating hormone (FSH) levels compared with the reference population. By contrast, no significant differences were observed in testicular volume or serum testosterone levels between the groups. The prevalence of urological comorbidities, including cryptorchidism, was higher in patients with TC but did not differ significantly from the reference population (Table 3).

**Table 3:** Subgroup analysis of pre-treatment testicular function in testicular cancer patients compared with the healthy reference population. Continuous variables are presented as mean (SD) and median (IQR; 25th–75th percentile). Categorical variables are presented as *n* (%). Group comparisons were performed using the chi-square test or Fisher’s exact test when appropriate. Statistically significant *P*-values (*P* < 0.05) are shown in bold.

	Reference population (*n* = 2,871)		Testicular cancer (*n* = 184)		*P*-value
	*n*	Values	*n*	Values	
Urological comorbidity					
Any*	2,554	614 (24.0%)	149	43 (28.9%)	0.183
Cryptorchidism	2,871	58 (2.0%)	184	7 (3.8%)	0.110^†^
Clinical examination					
Testis volume^‡^ (mL)	1,539		22		0.963
Mean ± SD		17.6 ± 4.4		17.8 ± 17	
Median (IQR)		17.5 (15–20)		13.7 (10.1–15.8)	
Hormonal assessment					
Testosterone (nmol/L)	724		21		0.114
Mean ± SD		16 ± 6.3		20.2 ± 11.6	
Median (IQR)		15.4 (11.7–19.6)		20.7 (10.6–25.7)	
FSH (IU/L)	444		13		**0.030**
Mean ± SD		3.6 ± 2.4		13.6 ± 14.6	
Median (IQR)		3.06 (2.2–4.3)		10.5 (3.8–18.8)	

* Reported or documented prior to participating in the study (listed in Supplementary Table 3).

^†^ Fisher’s exact test.

^‡^ Testicular volume was assessed either clinically using an orchidometer (reference population) or by ultrasound (cancer patients) and is reported as the mean of both testes when available, otherwise the available value from only one side.

FSH, follicular-stimulating hormone.

### Applied cancer therapies according to cancer type and risk of gonadotoxicity

Overall, chemotherapy and surgery were the most frequently used treatment modalities, and most patients received multimodal treatment (Fig. 5A, B, C, D and Supplementary Table 8). In TC, almost all patients underwent surgery (98.9%) and most received chemotherapy (78.4%). In HC, chemotherapy was predominant (95.8%), and this group also had the highest rates of immunotherapy, targeted therapy and stem cell transplantation. In OSCs, chemotherapy (86.6%) and surgery (86.2%) were the most common, while radiotherapy was used more than in TC and HC.

**Figure 5:**
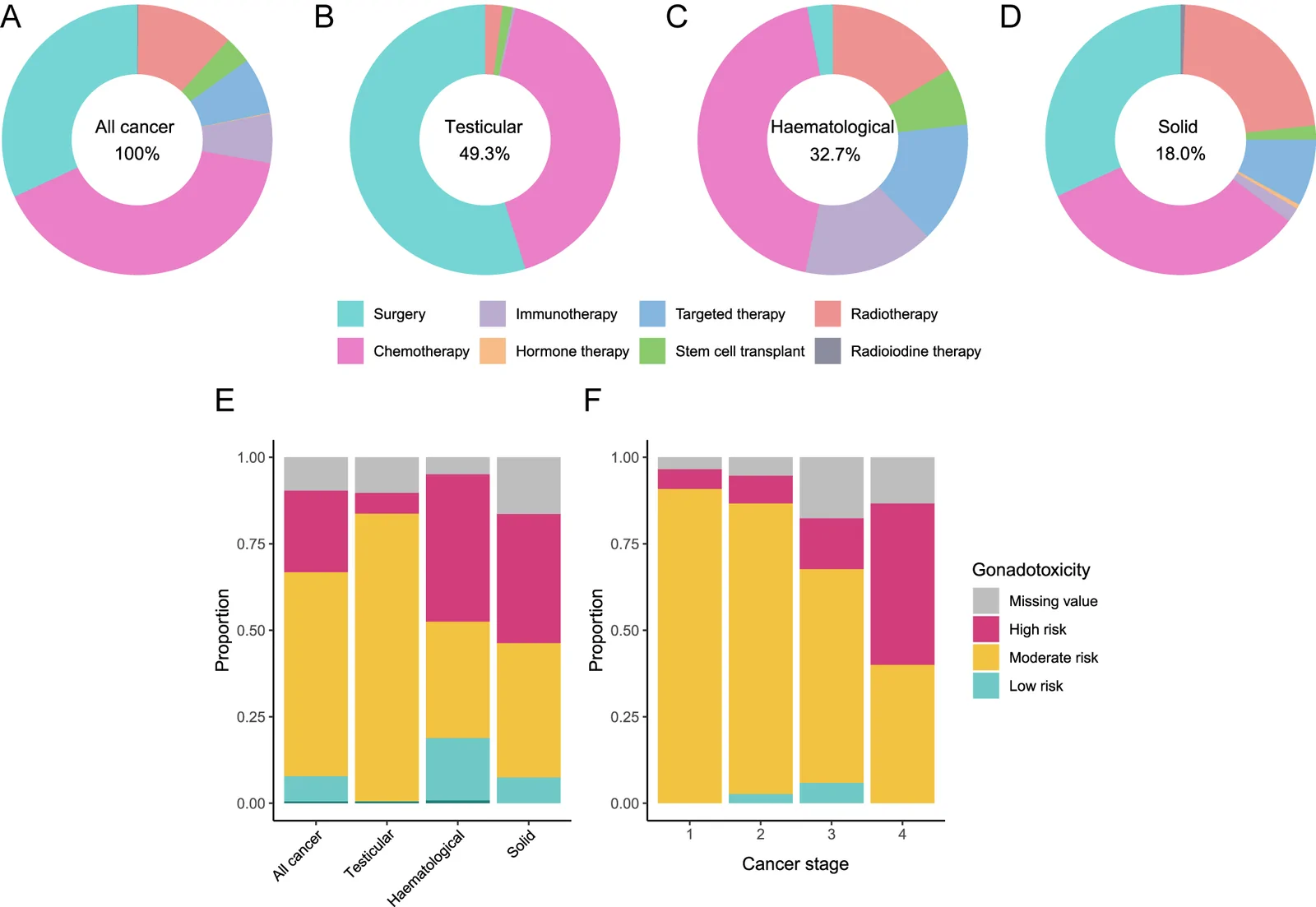
Distribution of cancer treatments and risk of gonadotoxicity according to cancer type and stage. (A, B, C, D) Doughnut charts showing the distribution of treatment modalities in all cancer patients and by cancer type (testicular, haematological and other solid cancers). Patients could receive multiple treatments; proportions, therefore, reflect treatment frequency rather than mutually exclusive categories. (E and F) Stacked bar charts illustrating gonadotoxicity risk by cancer type and stage. Risk was classified as low (<20%), moderate (20–80%) or high (>80%) based on the estimated risk of permanent azoospermia, using the highest-risk treatment when multiple therapies were administered. Proportions are shown within each group. Cancer stage was classified according to the Union Internationale Contre le Cancer (UICC) or the Ann Arbor system. The UICC classification applies to testicular cancer (stages 1–3) and other solid tumours (stages 1–4), while the Ann Arbor classification is used for Hodgkin and non-Hodgkin lymphomas (stages 1–4). Other haematological malignancies could not be categorised due to the lack of a unified classification system and were considered not applicable.

Most patients were exposed to therapies with at least moderate gonadotoxicity risk, with 59% classified as moderate risk and 23.6% as high risk (Fig. 5E and F and Supplementary Table 9). Moderate-risk treatment predominated in TC, mainly due to BEP-based chemotherapy (bleomycin, etoposide and cisplatin), whereas HC showed the highest proportion of high-risk exposures, largely reflecting intensive systemic treatment and stem cell transplantation. In OSCs, moderate- and high-risk treatments occurred in similar proportions. High-risk exposure increased with cancer stage and was consistently observed after stem cell transplantation.

Detailed results by specific cancer type are shown in Supplementary Table 10. Moderate gonadotoxicity risk predominated in TC with similar patterns in seminoma and non-seminoma, whereas high-risk treatments were more frequent in HC and selected OSC entities, particularly Hodgkin lymphoma, leukaemia, osteosarcoma, extragonadal germ cell tumours and brain cancer.

### Pre- and post-treatment changes in semen parameters

Changes in semen quality after treatment compared with before treatment are shown in Fig. 6 and Supplementary Table 11. Overall, azoospermia rates increased markedly after treatment across all cancer types. Among patients with TC, sperm concentration, TSC and TMSC showed similar counts after treatment, and semen volume remained stable. Total motility increased significantly. Despite this overall trend towards recovery of semen parameters, the proportion of azoospermia increased significantly after treatment. By contrast, patients with HC demonstrated a marked deterioration in semen parameters following treatment, with significant reductions in sperm concentration, TSC and TMSC, in accordance with the more gonadotoxic treatment regimens. Semen volume and total motility remained comparable.

**Figure 6:**
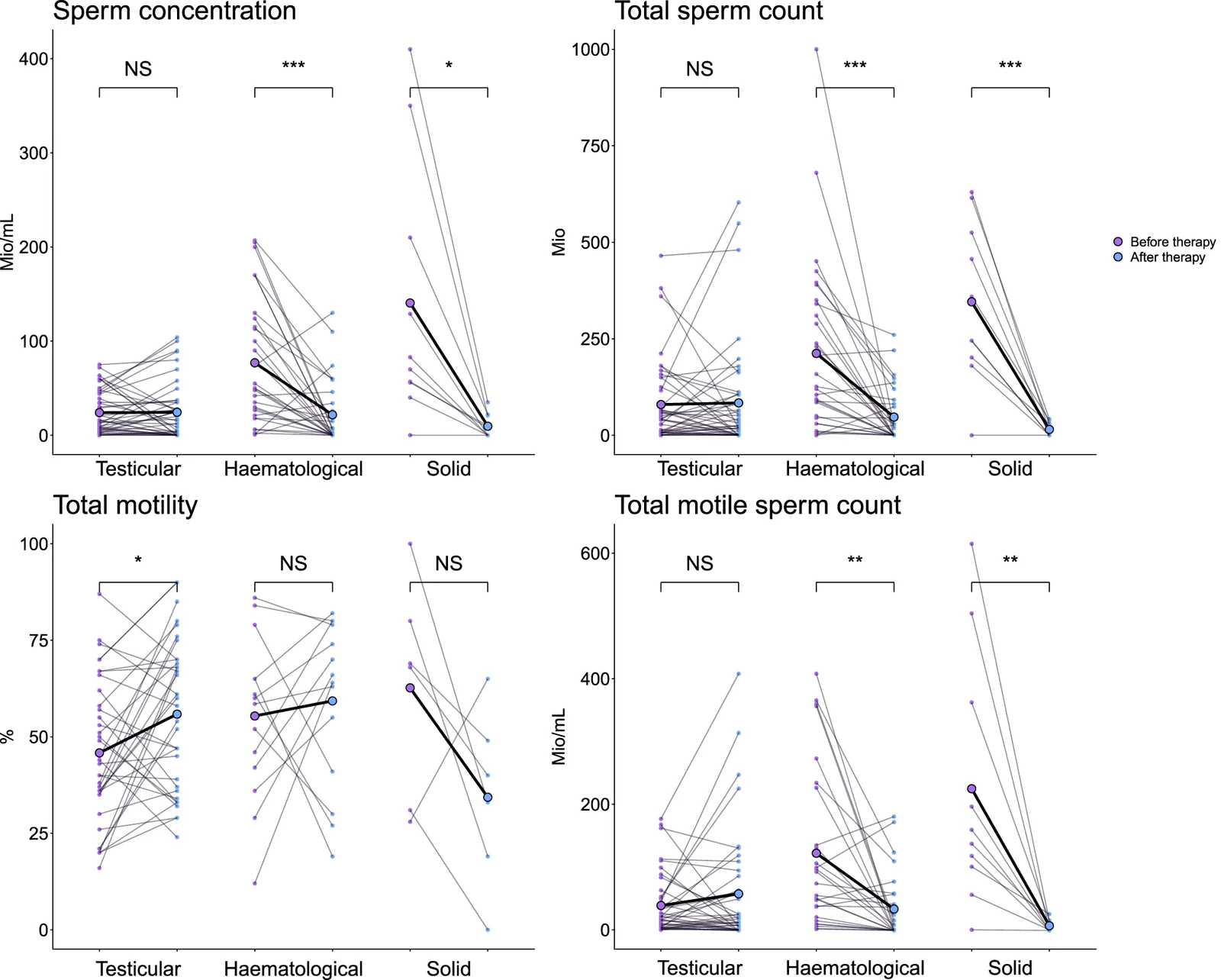
Pre- and post-treatment semen parameters according to cancer type. Paired plots showing individual changes in semen parameters before and after cancer treatment, stratified by cancer type (testicular, haematological and other solid cancers). Parameters include (A) sperm concentration (Mio/mL), (B) total sperm count (Mio), (C) total motility (%) and (D) total motile sperm count (Mio). Each grey line represents an individual patient, connecting pre- and post-treatment values. Larger coloured points indicate group means, connected by black lines. Post-treatment semen analysis was performed at a median of 20 months after completion of treatment. Total motility represents the proportion of all motile sperm (sum of fast progressive, slow progressive and non-progressive motility). Total motile sperm count was calculated as semen volume × sperm concentration × total motility. Statistical comparisons between pre- and post-treatment values were performed using paired *t*-tests. NS, not significant; *P < 0.05; **P < 0.01; ***P < 0.001.

Similarly, patients with OSCs showed significant reductions in sperm concentration, TSC and TMSC after treatment. Total motility and semen volume did not change. Due to the limited number of follow-up semen analyses after cancer treatment, a stratified analysis by tumour stage was not conducted.

### Fertility preservation and reproductive outcomes in cancer patients

Results on fertility preservation and reproductive outcomes are summarised in Table 4. On average, the first semen analysis for fertility preservation was performed 46.3 days after cancer diagnosis (median 26 days). Ejaculate cryopreservation was the most common fertility preservation method and was performed in most patients (93.2%) after the first semen sample. A second semen sample was cryopreserved in 20.1% of patients, most commonly due to low sperm count or a limited number of straws available for cryopreservation from the initial sample. However, ejaculate cryopreservation was not possible in 5.9% of patients due to azoospermia or severely reduced sperm concentration. TESE was performed in 2.9% of patients, with a success rate of 63.6%. TESE outcomes were reported descriptively only; all comparative analyses of semen parameters and regression models were restricted to ejaculated semen samples. Overall, 4.3% were unable to cryopreserve sperm by either ejaculation or TESE.

**Table 4:** Fertility preservation and reproductive outcomes in cancer patients. Continuous variables are presented as mean ± SD and median (IQR; 25th–75th percentile). Categorical variables are presented as *n* (%).

	All cancer (*n* = 373)		Testicular	Haematological	Solid
	*n*	Values			
*n* (%)			184 (49.3%)	122 (32.7%)	67 (18.0%)
Timing					
Time from cancer diagnosis to semen analysis before treatment (days)	94				
Mean ± SD		46.3 ± 45.2	56.1 ± 54.1	37.6 ± 33.4	28.1 ± 14
Median (IQR)		26 (16–53.8)	29 (15.8–102.5)	24.5 (16–40)	24 (19.2–36)
Time from end of cancer treatment* to semen analysis after treatment (months)	76				
Mean ± SD		42.5 ± 45.4	54 ± 55.8	34.3 ± 33.8	24.6 ± 13.6
Median (IQR)		20 (13–51.5)	24 (12.8–95.8)	18 (12.5–42.5)	18 (16–32)
Semen analysis after cancer treatment	373	95 (25.5%)	49 (26.6%)	36 (29.5%)	10 (14.9%)
Fertility preservation method					
Ejaculate analysis	373	373 (100%)	184 (100%)	122 (100%)	67 (100%)
Cryopreservation after first semen sample	370	345 (93.2%)	166 (91.7%)	115 (94.3%)	64 (95.5%)
Cryopreservation after second semen sample^†^	373	75 (20.1%)	39 (21.2%)	23 (18.9%)	13 (19.4%)
No cryopreservation with ejaculate	371	22 (5.9%)	14 (7.7%)	5 (4.1%)	3 (4.5%)
TESE	373	11 (2.9%)	10 (5.4%)	1 (0.8%)	0
Cryopreservation with TESE	373	7 (1.9%)	6 (3.3%)	1 (0.8%)	0
No cryopreservation with TESE	373	4 (1.1%)	4 (1.1%)	0	0
No cryopreservation with ejaculate or TESE	371	16 (4.3%)	9 (4.9%)	4 (3.3%)	3 (4.5%)
Family planning and fertility after cancer treatment					
Number of live births^‡^	343	100 (29.2%)	67 (40.1%)	22 (19.5%)	11 (17.5%)
Time to first child post-treatment (years)	72				
Mean ± SD		4.3 ± 3.4	4.1 ± 3.4	5 ± 3.7	3.9 ± 2.7
Median (IQR)		3.51 (1.9–5.7)	3.51 (1.9–5.7)	3.65 (3.1–6.4)	3.2 (2.2–4.4)
Fertility treatment^§^	372	29 (7.8%)	16 (8.7%)	10 (8.3%)	3 (4.5%)
ART	29	23 (79.3%)	12 (75.0%)	9 (90.0%)	2 (66.7%)
Clinical pregnancy with ART	23	17 (73.9%)	10 (83.3%)	6 (66.7%)	1 (50.0%)
Live births with ART	23	16 (69.6%)	10 (100%)	5 (83.3%)	1 (100%)
Miscarriage after ART	23	1 (4.3%)	0	1 (16.7%)	0

TESE, testicular sperm extraction; ART, assisted reproductive technology.

* Last documented cancer treatment.

^†^ A second semen sample was cryopreserved usually due to low sperm count or a limited number of straws available for cryopreservation from the initial sample.

^‡^ According to the Swiss National Civil Registry, irrespective of infertility or fertility treatment.

^§^ Fertility treatment includes cycle monitoring, monofollicular stimulation, inseminations and ART.

After completion of cancer treatment, the mean time to the next semen analysis was 42.5 months (median 20 months). Overall, 25.5% of patients underwent a post-treatment semen analysis in Lucerne.

In the whole cancer cohort, live birth after cancer treatment, irrespective of fertility treatment, was reported in 29.2% of patients. The highest proportion was observed among patients with TC (40.1%), whereas lower rates were seen in patients with HC (19.5%) and OSCs (17.5%). The mean time from the end of cancer treatment to the first child was 4.3 years, with longer intervals observed among patients with HC. Twenty-nine men (7.8%) returned after cancer treatment for fertility treatment. Of these, 23 (79.3%) underwent assisted reproductive technology (ART), resulting in 17 (73.9%) clinical pregnancies. Among all ART patients, 16 (69.6%) led to live births and one (4.3%) to a miscarriage. Only patients who underwent ART in Lucerne were considered in this analysis. Detailed data on reproductive outcomes, including mode of conception and infertility status, were not available for a large proportion of patients due to limitations inherent to the retrospective study design.

## Discussion

This is the first study analysing semen parameters before and after cancer treatment in a cohort of cancer patients undergoing fertility preservation in Switzerland with a comparison to a well-established reference population of young healthy men. Several important findings emerged. First, semen parameters were already reduced in cancer patients before the initiation of treatment in the unadjusted analyses. However, after adjustment for relevant confounding factors, this reduction remained significant only in patients with TC. This finding suggests that pre-treatment impairment of semen quality is particularly pronounced among men with testicular cancer, whereas the differences observed in the other cancer groups were substantially attenuated after accounting for differences in patient characteristics. The attenuation of several associations after multivariable adjustment indicates that demographic, lifestyle, and clinical characteristics contributed to the unadjusted differences between the cancer and reference populations. Furthermore, stratification reduced subgroup sizes, thereby decreasing statistical power, while residual clinical heterogeneity remained particularly within the HC and OSC groups. Grouping patients into broader diagnostic categories allowed clinically meaningful comparisons across major cancer groups; however, the remaining heterogeneity may have obscured more subtle tumour-specific associations. Therefore, the absence of statistically significant adjusted associations in these groups should not necessarily be interpreted as evidence of the absence of a cancer-related effect. In addition, TC patients showed the highest proportion of azoospermia, potentially reflecting a non-obstructive component as previously described (Huyghe & Chiu 2025). Azoospermia may have been underestimated due to incomplete archiving of semen analyses during the initial study period.

Second, the impact of cancer treatment on spermatogenesis differed substantially between cancer types, with systemic therapies in HC and OSCs showing a stronger detrimental effect than treatments for TC. Nevertheless, all cancer types showed a high prevalence of azoospermia after gonadotoxic treatment.

Third, despite increasing awareness of fertility preservation, follow-up semen analyses after treatment were performed in only a minority of patients. Beyond confirming previously reported patterns, a particular strength of our study is the comparison with a large population-based Swiss reference cohort, enabling a robust assessment of pre-treatment semen quality. In addition, the systematic classification of administered cancer treatments according to their estimated gonadotoxicity risk provides a clinically relevant framework for interpreting post-treatment changes in semen quality within the cancer cohort.

The observation that semen parameters may already be impaired before the initiation of cancer treatment has been reported previously and is particularly well described in patients with testicular cancer (Peluso *et al.* 2023). In our cohort, patients with TC showed the most pronounced reduction in semen parameters prior to treatment compared with the reference population. As semen volume and total motility in cancer patients were comparable to the reference population, the observed reduction in semen quality appears to be primarily attributable to quantitative alterations in sperm production. Baseline semen quality should be interpreted in the context of urological or endocrine comorbidities, such as cryptorchidism, which may impair spermatogenesis and fertility potential even before malignancy.

The subgroup analysis of testicular function in patients with TC showed significantly elevated FSH levels compared with the reference population, indicating pre-existing impaired spermatogenic function in this cancer type. Although hormonal data were available only for a small subset of patients and the analysis was, therefore, restricted to the TC subgroup, FSH and testosterone measurements provided complementary information on pre-treatment testicular function. The finding of elevated FSH in TC patients is consistent with impaired spermatogenic function; however, given the small and non-systematically assessed subgroup, these findings should be considered exploratory. More systematic prospective assessment of endocrine and semen parameters is needed to better distinguish pre-existing testicular dysfunction from treatment-related gonadotoxicity.

Changes in semen parameters following treatment varied across cancer types and likely reflect differences in treatment regimens and gonadotoxicity. Interestingly, although patients with TC exhibited the poorest semen quality parameters before treatment, they also appeared to have the greatest potential for recovery of spermatogenesis afterwards. Removal of the primary tumour may alleviate local testicular pathology and allow partial restoration of spermatogenic function in some patients. By contrast, patients with HC or OSCs are more frequently exposed to systemic therapies with higher gonadotoxic potential, which may cause sustained damage to spermatogonial stem cells and result in prolonged or permanent impairment of spermatogenesis. These observations underline the heterogeneous patterns of spermatogenic damage and recovery across cancer types and treatment modalities.

From a clinical perspective, it would be highly valuable to identify which patients are likely to recover spermatogenesis after treatment. However, reliable predictive markers remain limited and further research is required in this field.

The present results emphasise the importance of individualised fertility counselling before cancer treatment. While sperm cryopreservation remains the standard fertility preservation strategy for postpubertal males, its indication should be guided by the expected gonadotoxicity of the planned treatment and tailored to cancer type and stage, pre-existing comorbidities, patient preferences, and local legal and reimbursement frameworks. In Switzerland, health insurance covers fertility preservation for postpubertal patients up to 40 years of age undergoing cancer treatment with a moderate or high risk of persistent azoospermia. Storage costs are generally covered for five years and may be extended in selected cases (Federal Office of Public Health (FOPH) 2026).

An important observation in our cohort was the relatively low proportion of patients who underwent post-treatment semen analyses, with only about one-quarter having documented follow-up at Lucerne. This highlights a gap in survivorship care, as systematic assessment of reproductive function after cancer treatment remains inconsistent. However, the number of post-treatment conceptions observed in our cohort is encouraging and suggests that biological fatherhood remains achievable for many cancer survivors. Survivorship care should include not only oncological follow-up but also assessment of endocrine, metabolic, cardiovascular, bone and sexual health. Associated conditions such as hypogonadism, reduced libido, erectile dysfunction and psychological distress may significantly affect quality of life, highlighting the importance of integrated long-term care (Schover 2009, Halpern *et al.* 2020, Brannigan *et al.* 2021).

A major strength of this study is the availability of a Swiss reference population. This population-based dataset (Rahban *et al.* 2019, 2023) provides a more appropriate comparator for semen parameters than WHO reference values, which are derived from men who have already demonstrated fertility. This allows a more representative assessment of semen quality and reproductive potential in cancer patients. Furthermore, the availability of detailed information on potential confounders such as BMI, smoking, alcohol consumption and comorbidities enabled adjustment for important covariates and strengthened the validity of the analysis.

Nevertheless, several limitations should be considered. First, the retrospective design may have introduced information bias, as clinical data were extracted from medical records and some variables were incompletely documented, particularly in earlier years of the study period. However, inclusion of these older samples was important to maximise sample size and ensure adequate representation of patients across the study period. Furthermore, hormonal data were available for only a small, non-systematically assessed subgroup of patients and were predominantly derived from patients with TC, introducing potential selection bias, limiting the generalisability of the hormonal analyses and warranting cautious interpretation of these findings. Second, semen analyses were performed using different methodologies in the two populations: conventional manual microscopic evaluation according to WHO recommendations in cancer patients and computer-assisted sperm analysis (CASA) in the reference population. Although both approaches are widely used, methodological differences may affect comparability, particularly for motility parameters. Third, the cancer cohort was considerably smaller than the reference population. In addition, substantial heterogeneity within the cancer cohort remained with respect to tumour type, treatment modalities and the timing of post-treatment semen analyses, which may have limited the ability to detect tumour-specific effects despite stratification into broader diagnostic categories. In particular, differences in treatment exposure and follow-up intervals are likely to have influenced the extent of spermatogenic impairment and recovery observed after treatment, thereby limiting the comparability of post-treatment outcomes across cancer groups. In addition, the reference population consisted of Swiss military recruits who were younger than the cancer cohort. Although male reproductive functions generally decline later in life (Martins Da Silva & Anderson 2022) and age was adjusted for in the analyses, residual age-related confounding cannot be completely excluded. Finally, post-treatment follow-up data were limited, and the interval between treatment completion and follow-up semen analysis varied considerably, which may have influenced the assessment of spermatogenic recovery. Moreover, as post-treatment semen analyses were available for only a subset of patients, selection bias cannot be excluded, as patients returning for follow-up may not be representative of the entire cancer cohort.

## Further directions

Further research is needed to better understand long-term reproductive outcomes after cancer treatment. In a subsequent analysis, we aim to investigate reproductive outcomes in greater detail, including infertility, use of assisted reproductive technologies and reproductive success rates. Such data will provide a more comprehensive understanding of reproductive trajectories of male cancer survivors and help inform fertility counselling and survivorship care strategies. Future studies should seek to identify patients at greatest risk of treatment-related infertility who are most likely to benefit from fertility preservation strategies, as well as those with a lower expected risk of reproductive impairment.

## Conclusion

This study demonstrates that impaired semen quality may already be present in cancer patients prior to treatment and that the impact of cancer treatment on spermatogenesis varies substantially depending on cancer type and treatment regimen. Importantly, additional factors influencing spermatogenesis, such as endocrine or urological comorbidities, should also be considered when interpreting fertility potential. These findings highlight the need for individualised fertility counselling while underlining the importance of structured survivorship care that addresses reproductive health alongside other long-term consequences of cancer and its treatment.

## Supplementary materials



## Declaration of interest

R Rahban is an Associate Editor of *Reproduction *&* Fertility* and was not involved in the review or editorial process for this paper, on which she is listed as an author. The authors declare that there are no conflicts of interest.

## Funding

This work was supported by the Division of Reproductive Medicine of the Women’s Hospital at the Lucerne University Teaching and Research Hospital to MV and MS and by the Department of Genetic Medicine and Development at the University of Geneva to RR. This research did not receive any specific grant from any funding agency in the public, commercial or not-for-profit sector.

## Author contribution statement

MS, AKS, RR and MV conceived the study and designed the research. MS, RR, AS and MV collected and curated the data. CJ performed the statistical analyses. MS and MV drafted the manuscript. RR, BC, GT, AS, CJ, SN and AKS critically revised the manuscript. All authors approved the final version of the manuscript.
