# Supplementary material for: Semen quality in men with cancer: comparison with a healthy Swiss reference population
Source: Reprod Fertil. 2026 Oct 13;7(4):RAF260110. doi: 10.1530/RAF-26-0110 (PMC13695437; doi:10.1530/RAF-26-0110)
Supplement: Supplementary file 1 [file RAF-26-0110_supplementary_tables.pdf]

Supplementary material

Supplementary Table 1: Risk assessment of gonadotoxicity associated with cancer treatment

Treatment	Low Risk (<20%[1])	Moderate Risk (20-80%[1])	High Risk (>80%[1])	Unknown Risk
**Chemotherapy**	• Vincristine<br>• Methotrexate<br>• Bleomycin<br>• Vinblastine<br>• Etoposide<br>• Actinomycin D<br>• Mesna<br>• Idarubicin<br>• Daunorubicin<br>• Doxorubicin<br>• Cytarabine<br>• Azacitidine<br>• Cladribine<br>• 6-Mercaptopurine<br>• Hydroxycarbamide<br>• ABVD[2]<br>• R-CHOP[3], CHOP[4], CHEOP[5]<br>• COPDAC[6]	• Cyclophosphamide <5 g/m^2<br>• Ifosfamide <20.5 g/m^2<br>• Procarbazine <5.83 g/m^2<br>• Cisplatin <600 mg/m^2<br>• Carboplatin<br>• BEP[7]	• Cyclophosphamide >5 g/m^2<br>• Ifosfamide >20.5 g/m^2<br>• Procarbazine >5.83 g/m^2<br>• Cisplatin >600 mg/m^2<br>• Escalated BEACOPP[8]<br>• COPP[9]<br>• DA-EPOCH-R[10]	• Clofarabine<br>• Amsacrine<br>• Capecitabine<br>• Temozolomide<br>• FOLFOX[11]
**Radiotherapy**	• Locations excluding brain or testes	• Cranial <40 Gy[12]<br>• Pelvic with gonadal shielding<br>• Inguinal 30-40 Gy[12]	• Testicular >2 Gy[12]<br>• Cranial ≥40 Gy[12]	• N/A[13]
**Surgery**	• Partial orchiectomy with normal-sized contralateral testis	• Unilateral orchiectomy with normal-sized contralateral testis<br>• Nerve-sparing retroperitoneal lymphadenectomy<br>• Surgery on descending colon or rectum	• Bilateral orchiectomy<br>• Unilateral orchiectomy with atrophic contralateral testis<br>• Partial orchiectomy in a solitary testis<br>• Non-nerve-sparing retroperitoneal lymphadenectomy<br>• Surgery on hypothalamus or pituitary gland	• N/A[13]
**Immunotherapy**	• N/A[13]	• N/A[13]	• N/A[13]	• Atezolizumab<br>• Pembrolizumab<br>• Rituximab<br>• Brentuximab
**Targeted therapy**	• Imatinib<br>• Nilotinib<br>• Dasatinib	• N/A[13]	• N/A[13]	
**Stem cell transplantation**	• N/A[13]	• N/A[13]	• Stem Cell Transplantation	• N/A[13]
**Combinations**	• N/A[13]	• ABVD[2] + inguinal radiotherapy	• N/A[13]	• N/A[13]

[1] Risk of permanent azoospermia

[2] Doxorubicin, bleomycin, vinblastine, dacarbazine

[3] Rituximab, cyclophosphamide, hydroxydaunorubicin, vincristine, prednisolone

[4] Cyclophosphamide, hydroxydaunorubicin, vincristine, prednisolone

[5] Cyclophosphamide, hydroxydaunorubicin, etoposide, vincristine, prednisolone

[6] Cyclophosphamide, vincristine, prednisolone, dacarbazine

[7] Bleomycin, etoposide, cisplatin

[8] Bleomycin, etoposide, doxorubicin, cyclophosphamide, vincristine, procarbazine, prednisolone

[9] Cyclophosphamide, vincristine, procarbazine, prednisolone

[10] Dose-adjusted etoposide, prednisolone, vincristine, cyclophosphamide, hydroxydaunorubicin, rituximab

[11] Folinic acid, fluorouracil, oxaliplatin

[12] Gray

[13] Not applicable

References: (Dohle 2010; Chang *et al.* 2017; Schweizerische Gesellschaft für Gynäkologie und Geburtshilfe (SGGG) 2017; Lambertini *et al.* 2020; Brannigan *et al.* 2021; Rambhatla *et al.* 2021; Mauz-Körholz *et al.* 2022; Pagano *et al.* 2022; Himpe *et al.* 2023; Xue *et al.* 2023; Balcerek *et al.*)

Supplementary Table 2: List of cancer types in cancer patients

Testicular cancer
- Seminoma
- Non-seminoma
- Germ cell neoplasia in situ (GCNIS)
- Malignant Leydig cell tumour
- Not further specified testicular cancer due to incomplete medical records

Haematological cancer
- Hodgkin lymphoma
- Non-Hodgkin lymphoma
- Leukaemia
- Multiple myeloma
- Myelodysplastic syndrome

Other solid cancer
- Gastrointestinal cancer
- Brain and central nervous system (CNS) tumour
- Bone sarcoma
- Soft tissue sarcoma
- Extragonadal germ cell tumour
- Lung cancer
- Prostate cancer
- Bladder cancer
- Thyroid cancer
- Nasopharyngeal cancer
- Skin cancer
- Breast cancer

Supplementary Table 3: List of comorbidities (current condition or history)

Urological comorbidities
- Cryptorchidism
- Testicular torsion
- Orchiectomy or hemicastration
- Primary hypogonadism
- Varicocele
- Epididymitis
- Prostatitis
- Sexually transmitted disease
- Testicular trauma
- Penile fracture
- Vasectomy
- Erectile dysfunction
- Hydrocele
- Spermatocele
- Hypospadias
- Phimosis
- Circumcision
- Frenulotomy or frenuloplasty
- Benign prostatic hyperplasia
- Inguinal hernia (surgically treated)
- Mumps
- Other conditions that could not be assigned to a specific category

Endocrine comorbidites
- Diabetes
- Thyroid disease
- Adrenal disease
- Cushing syndrome
- Other conditions that could not be assigned to a specific category

Other comorbidites
- Genetic disorders
- Haematological disorders
- Rheumatological disorders
- Neurological disorders
- Infectious diseases (excluding sexually transmitted diseases)
- Cardiovascular diseases (excluding arterial hypertension and heart failure)
- Arterial hypertension
- Heart failure
- Hypercholesterolaemia
- Hepatic diseases
- Nephrological diseases
- Gastrointestinal diseases
- Pulmonary diseases
- Psychiatric disorders
- Musculoskeletal disorders
- Dermatological diseases
- Ophthalmological diseases
- Other conditions that could not be assigned to a specific category

Supplementary Table 4: Pre-treatment semen parameters in cancer patients according to cancer type compared with the healthy reference population

	**Reference population**			**Cancer patients**								
	**n=2871**				**All cancer (n=373)**		**Testicular (n=184, 49.3%)**		**Haematological (n=122, 32.7%)**		**Solid (n=67, 18.0%)**	
	N with data	Mean (SD)	Median (Q1-Q3)	N with data	Mean (SD)	Median (Q1-Q3)	Mean (SD)	Median (Q1-Q3)	Mean (SD)	Median (Q1-Q3)	Mean (SD)	Median (Q1-Q3)
**Semen parameters**												
Ejaculation abstinence[1] (days)	2871	3.7 (13.9)	2.86 (2 - 3.8)	314	4.9 (7.7)	3 (2 - 5)	4.4 (5.2)	3 (2 - 5)	5.8 (10.2)	4 (2 - 5)	4.7 (8)	3 (2 - 6)
Volume (mL)	2871	3 (1.4)	2.8 (2 - 3.8)	342	2.9 (1.7)	2.6 (1.7 - 4)	3.1 (1.7)	3 (2 - 4)	2.6 (1.6)	2.5 (1.5 - 3.5)	2.8 (1.7)	2.5 (1.5 - 3.8)

Sperm Concentration (Mio/mL)	2871	65.6 (66.1)	47.67 (22.1 - 87.5)	343	47.3 (55.1)	30 (9 - 69)	30.3 (33.5)	20 (5.8 - 47.5)	58.8 (56.7)	42 (14 - 77)	73.4 (79.6)	50 (23.5 - 81.5)
Total Sperm Count (Mio)	2871	186.9 (218.6)	126.82 (59.6 - 249.4)	340	139.6 (178.7)	74.5 (18.5 - 187.5)	100.5 (132.1)	50 (14 - 139.1)	161.2 (189.4)	88 (23.8 - 238)	210 (237.8)	160 (39 - 265)
Total motility[2] (%)	2838	53.4 (18.4)	53.81 (40.7 - 66.5)	314	54.4 (21.8)	58 (41 - 70)	54.6 (22.1)	55 (40 - 70)	53.2 (21.3)	58 (40 - 69)	56.2 (22.3)	60.5 (43.8 - 69.5)
TMSC[3] (Mio)	2838	105.6 (123.9)	67.04 (28.4 - 142.8)	311	83.7 (112.5)	40.95 (12 - 110.3)	61.4 (84.3)	31.92 (10.9 - 79.6)	97.4 (121.8)	52 (14.4 - 121.6)	121.3 (147.4)	58.5 (19.9 - 158.8)

Continuous variables are presented as mean (SD) and median (IQR; 25th-75th percentile). Categorical variables are presented as n (%).

[1] Abstinence period refers to the number of days between the last ejaculation and semen sample collection

[2] All motile sperm (a + b + c); a = fast progressive movement, b = slow progressive movement, c = non-progressive movement

[3] Total motile sperm count: volume × sperm concentration × total motility (a + b + c)

Supplementary Table 5: Pre-treatment semen parameters in cancer patients according to cancer stage[1] compared with the healthy reference population

	Reference population (n=2871)			Cancer patients All stages (n=302)			Stage 1 (n=98, 32.5%)		Stage 2 (n=109, 36.1%)		Stage 3 (n=56, 18.5%)		Stage 4 (n=39, 12.9%)	
	N with data	Mean (SD)	Median (Q1-Q3)	N with data	Mean (SD)	Median (Q1-Q3)	Mean (SD)	Median (Q1-Q3)	Mean (SD)	Median (Q1-Q3)	Mean (SD)	Median (Q1-Q3)	Mean (SD)	Median (Q1-Q3)
**Semen parameters**														
Ejaculation abstinence[2] (days)	2871	3.7 (13.9)	2.86 (2 - 3.8)	264	4.9 (7.9)	3 (2 - 5)	3.5 (2.7)	3 (2 - 4)	5.6 (10.1)	4 (2 - 5)	5.4 (8.9)	3 (2 - 6)	5.5 (8)	3 (2 - 6)
Volume (mL)	2871	3 (1.4)	2.8 (2 - 3.8)	278	3.0 (1.8)	2.8 (1.8 - 4)	3.1 (1.8)	3 (1.9 - 4)	3.3 (1.8)	3 (2 - 4.1)	2.7 (1.6)	2.3 (1.6 - 3.5)	2.3 (1.4)	2.2 (1.1 - 2.9)
Sperm Concentration (Mio/mL)	2871	65.6 (66.1)	47.67 (22.1 - 87.5)	281	45.5 (52.5)	29 (9 – 63.5)	39.4 (46.5)	24 (10 - 56)	38.4 (42.9)	22 (7.8 - 60)	58.9 (66.7)	39 (16 - 73)	62.3 (63.3)	49 (13.2 - 72.2)
Total Sperm Count (Mio)	2871	186.9 (218.6)	126.82 (59.6 - 249.4)	279	139.5 (182.3)	75.0 (17.2 - 180.0)	127.9 (170.4)	67.5 (14 - 168)	137.6 (172.9)	62.95 (13.5 - 186)	168.9 (235.9)	80.5 (31.8 - 233.8)	132 (150)	88 (22.5 - 161)
Total motility[3] (%)	2838	53.4 (18.4)	53.81 (40.7 - 66.5)	254	53.9 (21.6)	57 (40 - 69)	55.4 (22.1)	58 (42.2 - 71.2)	52 (22.2)	54 (37.2 - 66.4)	56.6 (20.2)	58 (44 - 72)	52.7 (20.9)	58.5 (38 - 68)
TMSC[4] (Mio)	2838	105.6 (123.9)	67.04 (28.4 - 142.8)	251	84.6 (114.4)	37.96 (11.5 - 111.4)	76.6 (97.3)	37.84 (11.3 - 93.6)	84.4 (121.2)	36 (7.2 - 112.5)	109.8 (141.7)	48.6 (21.4 - 130.6)	73.2 (92.2)	39.33 (13.2 - 92.6)

Continuous variables are presented as mean (SD) and median (IQR; 25th-75th percentile). Categorical variables are presented as n (%).

[1] Cancer stage was classified according to the Union Internationale Contre le Cancer (UICC) or the Ann Arbor system. The UICC classification applies to testicular cancer (stages 1-3) and other solid tumours (stages 1-4), while the Ann Arbor classification is used for Hodgkin and non-Hodgkin lymphomas (stages 1-4). Other haematological malignancies could not be categorised due to the lack of a unified classification system and were considered not applicable.

[2] Abstinence period refers to the number of days between the last ejaculation and semen sample collection

[3] All motile sperm (a + b + c); a = fast progressive movement, b = slow progressive movement, c = non-progressive movement

[4] Total motile sperm count: volume × sperm concentration × total motility (a + b + c)

Supplementary Table 6: Pre-treatment rate of azoospermia in cancer patients according to cancer type compared with the healthy reference population

	Reference population	Cancer patients			
	n=2871	All cancer (n=373)	Testicular (n=184, 49.3%)	Haematological (n=122, 32.7%)	Solid (n=67, 18.0%)
**Azoospermia**	30/2868 (1.0%)	10/324 (3.1%)	7/164 (4.3%)	3/104 (2.9%)	0/56 (0%)

Supplementary Table 7: Unadjusted and adjusted linear regression analyses of pre-treatment semen parameters in cancer patients stratified by cancer type, compared with the healthy reference population

Adjusted[1] coefficients

	Volume (mL)			TMSC[2] (Mio)			Concentration (Mio/mL)			Total sperm count (Mio)			Total motility[3] (%)		
	β[4]	95% CI[5]	p-value	β[4]	95% CI[5]	p-value	β[4]	95% CI[5]	p-value	β[4]	95% CI[5]	p-value	β[4]	95% CI[5]	p-value
**Group**															
Reference population	(ref)			(ref)			(ref)			(ref)			(ref)		
Testicular	0.08	-0.43 to 0.59	0.747	-53.34	-84.57 to -22.12	**0.001**	-38.07	-53.61 to -22.52	**<0.001**	-92.51	-143.59 to -41.42	**<0.001**	2.06	-5.01 to 9.13	0.568
Haematological	-0.87	-1.37 to-0.38	**0.001**	-9.50	-61.08 to 42.09	0.718	2.91	-21.82 to 27.64	0.818	-23.69	-105.48 to 58.10	0.570	0.30	-6.04 to 6.64	0.925
Solid	-0.64	-1.29 to 0.00	0.051	24.13	-37.16 to 85.41	0.440	22.58	-13.40 to 58.56	0.219	45.84	-36.08 to 127.77	0.273	1.33	-8.34 to 11.01	0.787
**Age** (years)	0.03	0.00 to 0.07	**0.026**	1.03	-1.64 to 3.69	0.450	0.07	-1.34 to 1.49	0.920	0.73	-3.43 to 4.89	0.731	0.07	-0.44 to 0.57	0.786
**Cryptorchidism**															
No	(ref)			(ref)			(ref)			(ref)			(ref)		
Yes	0.02	-0.28 to 0.32	0.912	-26.54	-50.79 to -2.29	**0.032**	-16.92	-31.03 to - 2.80	**0.019**	-46.63	-87.76 to -5.50	**0.026**	-6.54	-11.87 to - 1.20	**0.016**
**Endocrine comorbidities[6]**															
No	(ref)			(ref)			(ref)			(ref)			(ref)		
Yes	-0.78	-1.39 to -0.17	**0.013**	-63.33	-86.32 to -40.34	**<0.001**	-28.60	-42.96 to - 14.24	**<0.001**	-102.04	-145.73 to - 58.34	**<0.001**	-4.11	-15.52 to 7.31	0.481
**Nicotine use**															
No or former	(ref)			(ref)			(ref)			(ref)			(ref)		
Occasional or active	0.03	-0.12 to 0.17	0.712	-2.76	-14.23 to 8.71	0.637	-4.82	-10.97 to 1.33	0.125	-10.65	-30.48 to 9.19	0.293	2.19	0.30 to 4.09	**0.023**
**Alcohol consumption**															
Never	(ref)			(ref)			(ref)			(ref)			(ref)		
Sometimes or often	-0.02	-0.17 to 0.12	0.740	-2.56	-14.74 to 9.61	0.680	0.23	-6.36 to 6.82	0.946	-3.13	-25.94 to 19.68	0.788	-0.39	-2.27 to 1.49	0.684
**Ejaculation abstinence[7]** (days)															
[0-2[	(ref)			(ref)			(ref)			(ref)			(ref)		

[2-7]	0.65	0.53 to 0.78	**<0.001**	32.41	22.78 to 42.04	**<0.001**	8.38	2.39 to 14.38	**0.006**	60.27	43.41 to 77.14	**<0.001**	-0.96	-2.73 to 0.80	0.283
>7	1.13	0.81 to 1.46	**<0.001**	112.94	69.89 to 155.99	**<0.001**	49.48	26.93 to 72.02	**<0.001**	269.16	161.16 to 376.55	**<0.001**	-6.30	-10.77 to -1.83	**0.006**
**BMI[8]**	-0.01	-0.03 to 0.01	0.192	-0.43	-2.02 to 1.16	0.596	-0.14	-1.02 to 0.74	0.755	-2.29	-5.10 to 0.53	0.111	0.29	0.06 to 0.53	**0.016**

Statistically significant p-values (p < 0.05) are shown in bold.

[1] Adjustment was made on age, cryptorchidism, endocrine comorbidities, nicotine use, alcohol consumption, days of abstinence and body mass index

[2] Total motile sperm count: volume × sperm concentration × total motility (a + b + c)

[3] All motile sperm (a + b + c); a = fast progressive movement, b = slow progressive movement, c = non-progressive movement

[4] Computed from linear regressions and the robust Huber-White (HC3) method

[5] Confidence interval

[6] Reported or documented prior to participating in the study (diabetes, thyroid disease, adrenal disease, Cushing syndrome, other)

[7] Abstinence period refers to the number of days between the last ejaculation and semen sample collection

[8] Body mass index

Unadjusted coefficients

	**Volume (mL)**			**TMSC[1] (Mio)**			**Concentration (Mio/mL)**			**Total sperm count (Mio)**			**Total motility[2] (%)**		
	**β[3]**	**95% CI[4]**	**p-value**	**β[3]**	**95% CI[4]**	**p-value**	**β[3]**	**95% CI[4]**	**p-value**	**β[3]**	**95% CI[4]**	**p-value**	**β[3]**	**95% CI[4]**	**p-value**
**Group**															
Reference population	(ref)			(ref)			(ref)			(ref)			(ref)		
Testicular	0.42	0.01 to 0.83	**0.045**	-43.56	-63.02 to -24.10	**<0.001**	-38.20	-46.07 to -30.34	**<0.001**	-88.29	-120.44 to -56.14	**<0.001**	2.91	-2.71 to 8.53	0.310
Haematological	-0.53	-1.03 to -0.02	**0.040**	12.19	-33.90 to 58.27	0.604	9.06	-12.01 to 30.13	0.399	11.33	-60.49 to 83.14	0.757	0.45	-5.96 to 6.86	0.890
Solid	-0.36	-0.95 to 0.23	0.232	32.76	-28.75 to 94.27	0.296	22.75	-11.85 to 57.35	0.197	46.63	-31.42 to 124.68	0.242	2.04	-6.67 to 10.76	0.646

Statistically significant p-values (p < 0.05) are shown in bold.

[1] Total motile sperm count: volume × sperm concentration × total motility (a + b + c)

[2] All motile sperm (a + b + c); a = fast progressive movement, b = slow progressive movement, c = non-progressive movement

[3] Computed from linear regressions and the robust Huber-White (HC3) method

[4] Confidence interval

Supplementary Table 8: Applied treatment[1] in cancer patients according to cancer type

**Treatment[1]**	**N with data**	**All cancer (n=373)**	**Testicular (n=184, 49.3%)**	**Haematological (n=122, 32.7%)**	**Solid (n=67, 18.0%)**
Surgery	370	246 (66.5%)	182 (98.9%)	8 (6.6%)	56 (86.2%)
Chemotherapy	363	311 (85.7%)	138 (78.4%)	115 (95.8%)	58 (86.6%)
Immunotherapy	369	45 (12.2%)	1 (0.5%)	41 (34.7%)	3 (4.5%)
Hormone therapy	373	1 (0.3%)	0	0	1 (1.5%)

Targeted therapy	369	52 (14.1%)	0	38 (32.2%)	14 (20.9%)
Stem cell transplant	369	25 (6.8%)	4 (2.2%)	18 (15.3%)	3 (4.5%)
Radiotherapy	366	90 (24.6%)	7 (3.8%)	43 (36.4%)	40 (61.5%)
Radioiodine therapy	373	1 (0.3%)	0	0	1 (1.5%)

[1] Combinations/multiple cancer therapies possible

Supplementary Table 9: Risk of gonadotoxicity[1] in cancer patients according to cancer type, stage and applied treatment[2]

	N with data	No known risk (n=2, 0.5%)	Low risk[3] (n=27, 7.2%)	Moderate risk[4] (n=220, 59.0%)	High risk[5] (n=88, 23.6%)	Unknown (n=36, 9.7%)
**Cancer type**						
Testicular	184	1 (0.5%)	0	153 (83.2%)	11 (6.0%)	19 (10.3%)
Haematological	122	1 (0.8%)	22 (18.0%)	41 (33.6%)	52 (42.6%)	6 (4.9%)
Solid	67	0	5 (7.5%)	26 (38.8%)	25 (37.3%)	11 (16.4%)
**Cancer stage[6]**						
1	87	0	0	79 (90.8%)	5 (5.7%)	3 (3.4%)
2	75	0	2 (2.7%)	63 (84.0%)	6 (8.0%)	4 (5.3%)
3	34	0	2 (5.9%)	21 (61.8%)	5 (14.7%)	6 (17.6%)
4	15	0	0	6 (40.0%)	7 (46.7%)	2 (13.3%)
**Treatment[2]**						
Surgery	246	0	6 (2.4%)	177 (72.0%)	34 (13.8%)	29 (11.8%)
Chemotherapy	311	0	22 (7.1%)	183 (58.8%)	83 (26.7%)	23 (7.4%)
Immunotherapy	45	0	1 (2.2%)	23 (51.1%)	19 (42.2%)	2 (4.4%)
Hormone therapy	1	0	0	1 (100%)	0	0
Targeted therapy	52	0	1 (1.9%)	26 (50.0%)	21 (40.4%)	4 (7.7%)
Stem cell transplant	25	0	0	0	25 (100%)	0
Radiotherapy	90	0	15 (16.7%)	38 (42.2%)	37 (41.1%)	0
Radioiodine therapy	1	0	1 (100%)	0	0	0

[1] Gonadotoxicity was assessed for each cancer patient according to **Supplementary Table 1**. If multiple therapies were applied, the one with the higher risk score was chosen

[2] Combinations/multiple cancer therapies were possible

[3] Risk of permanent azoospermia <20%

[4] Risk of permanent azoospermia 20-80%

[5] Risk of permanent azoospermia >80%

[6] Cancer stage was classified according to the Union Internationale Contre le Cancer (UICC) or the Ann Arbor system. The UICC classification applies to testicular cancer (stages 1-3) and other solid tumours (stages 1-4), while the Ann Arbor classification is used for Hodgkin and non-Hodgkin lymphomas (stages 1-4). Other haematological malignancies could not be categorised due to the lack of a unified classification system and were considered not applicable.

Supplementary Table 10: Risk of gonadotoxicity[1] in cancer patients according to specific cancer type

Specific cancer type	N with data (%[2])	No known risk (n=2, 0.5%)	Low risk[3] (n=27, 7.2%)	Moderate risk[4] (n=220, 59.0%)	High risk[5] (n=88, 23.6%)	Unknown (n=36, 9.7%)
**Testicular Cancer**						
Seminoma	59 (15.8%)	0	0	55 (93.2%)	2 (3.4%)	2 (3.4%)
Non-seminoma	105 (28.2%)	0	0	91 (86.7%)	7 (6.7%)	7 (6.7%)
Other testicular cancer[6]	20 (5.4%)	1 (5.0%)	0	7 (35.0%)	2 (10.0%)	10 (50.0%)
**Haematological Cancer**						
Hodgkin lymphoma	65 (17.4%)	1 (1.5%)	13 (20.0%)	21 (32.3%)	28 (43.1%)	2 (3.1%)
Non-Hodgkin lymphoma	35 (9.4%)	0	4 (11.4%)	18 (51.4%)	12 (34.3%)	1 (2.9%)
Leukaemia	20 (5.4%)	0	5 (25.0%)	1 (5.0%)	11 (55.0%)	3 (15.0%)
Other haematological cancer[6]	2 (0.5%)	0	0	1 (50%)	1 (50%)	0
**Solid Tumours**						
Intestinal cancer	17 (4.5%)	0	1 (5.9%)	9 (52.9%)	0	7 (41.2%)
Osteosarcoma	10 (2.7%)	0	0	4 (40.0%)	6 (60.0%)	0
Soft tissue sarcoma	9 (2.4%)	0	2 (22.2%)	4 (44.4%)	3 (33.3%)	0
Brain cancer	12 (3.2%)	0	1 (8.3%)	2 (16.7%)	8 (66.7%)	1 (8.3%)
Extragonadal germ cell tumours	12 (3.2%)	0	0	3 (25.0%)	8 (66.7%)	1 (8.3%)
Other solid tumours[6]	7 (1.9%)	0	1 (14.3%)	4 (57.1%)	0	2 (28.6%)

[1] Gonadotoxicity was assessed for each cancer patient according to **Supplementary Table 1**. If multiple therapies were applied, the one with the higher risk score was chosen

[2] Percentage refers to all cancer patients (n=373)

[3] Risk of permanent azoospermia <20%

[4] Risk of permanent azoospermia 20-80%

[5] Risk of permanent azoospermia >80%

[6] Listed in **Supplementary Table 2**

Supplementary Table 11: Pre- and post-treatment[1] semen parameters according to cancer type

Cancer type	Variables	N with data	Pre-treatment	Post-treatment	Difference [95%CI[2]]	p-value
**Testicular**	Sperm concentration (Mio/mL)	44	24.1 (23.1)	24.5 (30.1)	0.4 [-7.9 to 8.7]	0.926
	Total sperm count (Mio)	44	79.7 (106.8)	83.9 (141.1)	4.2 [-35.0 to 43.4]	0.830
	Total motility[3] (%)	36	45.8 (18.6)	55.9 (19.2)	10.0 [2.6 to 17.5]	**0.010**
	TMSC[4] (Mio)	41	38.7 (48.4)	57.3 (92.0)	18.6 [-7.2 to 44.4]	0.153
	Volume (mL)	45	3.1 (1.8)	2.9 (1.7)	-0.2 [-0.7 to 0.2]	0.340
	Azoospermia	44	1/44 (2.3%)	8/44 (18.2%)	0.16 [0.09 to 0.32]	**<0.001**

**Haematological**	Sperm concentration (Mio/mL)	31	77.0 (66.6)	21.8 (34.4)	-55.2 [-80.3 to -30.1]	**<0.001**
	Total sperm count (Mio)	31	212.0 (222.5)	47.2 (71.2)	-164.8 [-246.3 to -83.3]	**<0.001**
	Total motility[3] (%)	14	55.4 (21.1)	59.3 (21.6)	3.9 [-13.6 to 21.4]	0.639
	TMSC[4] (Mio)	27	122.0 (128.4)	33.1 (54.0)	-88.8 [-141.0 to -36.7]	**0.002**
	Volume (mL)	31	2.8 (1.8)	2.5 (1.5)	-0.3 [-0.7 to 0.1]	0.171
	Azoospermia	27	0/27 (0%)	13/27 (48.1%)	0.48 [0.33 to 0.70]	**<0.001**
**Solid**	Sperm concentration (Mio/mL)	10	140.5 (138.9)	9.6 (12.4)	-130.9 [-222.4 to -39.4]	**0.010**
	Total sperm count (Mio)	10	345.7 (207.1)	15.2 (17.9)	-330.5 [-473.0 to -188.1]	**0.001**
	Total motility[3] (%)	6	62.7 (28.2)	34.3 (22.8)	-28.3 [-67.3 to 10.7]	0.121
	TMSC[4] (Mio)	10	224.8 (202.2)	6.4 (9.3)	-218.4 [-361.5 to -75.3]	**0.007**
	Volume (mL)	10	3.1 (1.9)	3.1 (2.5)	0.0 [-1.7 to 1.7]	0.979
	Azoospermia	10	0/10 (0%)	4/10 (40.0%)	0.40 [0.20 to 0.80]	**<0.001**

P-values were computed using paired t-tests for continuous variables and the bootstrap method for the paired proportions. Continuous variables are represented as mean (SD), and binary variables are represented as count (percentage). Statistically significant p-values ($p < 0.05$) are shown in bold.

[1] Semen analysis after treatment was conducted at a median of 20 months after cancer treatment

[2] Confidence interval

[3] All motile sperm (a + b + c); a = fast progressive movement, b = slow progressive movement, c = non-progressive movement

[4] Total motile sperm count: volume × sperm concentration × total motility (a + b + c)

Supplementary Table 12: Pre-treatment rate of azoospermia in cancer patients according to cancer stage[1] compared with the healthy reference population

	**Reference population**		**Cancer patients**					
	N with data	**(n=2871)**	N with data	**All stages (n=302)**	**Stage 1 (n=98, 32.5%)**	**Stage 2 (n=109, 36.1%)**	**Stage 3 (n=56, 18.5%)**	**Stage 4 (n=39, 12.9%)**
**Azoospermia**	2868	30 (1.0%)	263	9 (3.4%)	3 (3.5%)	3 (3.0%)	2 (4.7%)	1 (2.9%)

[1] Cancer stage was classified according to the Union Internationale Contre le Cancer (UICC) or the Ann Arbor system. The UICC classification was applies to testicular cancer (stages 1-3) and other solid tumours (stages 1-4), while the Ann Arbor classification is used for Hodgkin and non-Hodgkin lymphomas (stages 1-4). Other haematological malignancies could not be categorised due to the lack of a unified classification system and were considered not applicable.

Supplementary Table 13: Unadjusted and adjusted linear regression analyses of pre-treatment semen parameters of cancer patients and the healthy reference population

Unadjusted coefficients

	**Volume (mL)**			**TMSC[1] (Mio)**			**Concentration (Mio/mL)**			**Total sperm count (Mio)**			**Total motility[2] (%)**		
	**β[3]**	**95% CI[4]**	**p-value**	**β[3]**	**95% CI[4]**	**p-value**	**β[3]**	**95% CI[4]**	**p-value**	**β[3]**	**95% CI[4]**	**p-value**	**β[3]**	**95% CI[4]**	**p-value**
**Group**															
Reference population	(ref)			(ref)			(ref)			(ref)			(ref)		
Cancer patients	-0.08	-0.37 to 0.21	0.609	-7.22	-30.55 to 16.11	0.544	-8.76	-20.85 to 3.33	0.155	-24.21	-58.80 to 10.38	0.170	1.91	-1.90 to 5.72	0.325
**Age** (years)	0.02	-0.01 to 0.04	0.174	-0.12	-2.01 to 1.77	0.900	-0.63	-1.65 to 0.39	0.224	-1.55	-4.46 to 1.35	0.294	0.17	-0.22 to 0.55	0.396
**Cryptorchidism**															
No	(ref)			(ref)			(ref)			(ref)			(ref)		
Yes	0.03	-0.30 to 0.35	0.873	-31.97	-56.12 to -7.81	**0.010**	-19.96	-33.82 to -6.10	**0.005**	-58.82	-99.75 to -17.88	**0.005**	-6.01	-11.39 to -0.63	**0.029**
**Endocrine comorbidities[5]**															
No	(ref)			(ref)			(ref)			(ref)			(ref)		
Yes	-0.79	-1.55 to -0.03	**0.042**	-79.84	-93.41 to -66.28	**<0.001**	-39.69	-52.15 to -27.24	**<0.001**	-136.86	-162.58 to -111.15	**<0.001**	-2.99	-14.64 to 8.66	0.615
**Nicotine use**															
No or former	(ref)			(ref)			(ref)			(ref)			(ref)		
Occasional or active	-0.05	-0.20 to 0.10	0.521	-8.31	-19.80 to 3.17	0.156	-6.78	-12.82 to -0.75	**0.028**	-22.64	-42.32 to -2.95	**0.024**	2.41	0.57 to 4.25	**0.010**
**Alcohol consumption**															
Never	(ref)			(ref)			(ref)			(ref)			(ref)		
Sometimes or often	-0.04	-0.18 to 0.10	0.574	-7.00	-18.95 to 4.96	0.251	-2.31	-8.77 to 4.15	0.483	-14.12	-35.93 to 7.68	0.204	-0.02	-1.85 to 1.81	0.981
**Ejaculation abstinence[6]** (days)															
[0-2[	(ref)			(ref)			(ref)			(ref)			(ref)		
[2-7]	0.65	0.53 to 0.77	**<0.001**	32.74	23.23 to 42.25	**<0.001**	8.60	2.68 to 14.52	**0.004**	61.16	44.69 to 77.64	**<0.001**	-1.17	-2.92 to 0.59	0.192
>7	1.12	0.78 to 1.45	**<0.001**	113.84	71.58 to 156.10	**<0.001**	50.07	27.80 to 72.35	**<0.001**	270.68	164.61 to 376.74	**<0001**	-6.36	-10.78 to -1.94	**0.005**
**BMI[7]**	-0.01	-0.03 to 0.01	0.223	-0.74	-2.34 to 0.86	0.365	-0.41	-1.28 to 0.46	0.355	-3.12	-5.89 to -0.35	**0.028**	0.34	0.10 to 0.57	**0.005**

Statistically significant p-values ($p < 0.05$) are shown in bold.

[1] Total motile sperm count: volume × sperm concentration × total motility (a + b + c)

[2] All motile sperm (a + b + c); a = fast progressive movement, b = slow progressive movement, c = non-progressive movement

[3] Computed from linear regressions and the robust Huber-White (HC3) method

[4] Confidence interval

[5] Reported or documented prior to participating in the study (diabetes, thyroid disease, adrenal disease, Cushing syndrome, other)

[6] Abstinence period refers to the number of days between the last ejaculation and semen sample collection

[7] Body mass index

Adjusted[1] coefficients

	Volume (mL)			TMSC[2] (Mio)			Concentration (Mio/mL)			Total sperm count (Mio)			Total motility[3] (%)		
	β[4]	95% CI[5]	p-value	β[4]	95% CI[5]	p-value	β[4]	95% CI[5]	p-value	β[4]	95% CI[5]	p-value	β[4]	95% CI[5]	p-value
**Group**															
Reference population	(ref)			(ref)			(ref)			(ref)			(ref)		
Cancer patients	-0.45	-0.83 to -0.07	**0.020**	-17.11	-47.31 to 13.09	0.267	-8.25	-24.96 to 8.45	0.333	-32.61	-79.07 to 13.84	0.169	1.26	-3.78 to 6.31	0.624
**Age (years)**	0.04	0.01 to 0.07	**0.010**	0.64	-1.94 to 3.23	0.625	-0.19	-1.60 to 1.23	0.797	0.23	-3.80 to 4.27	0.820	0.08	-0.42 to 0.57	0.761
**Cryptorchidism**															
No	(ref)			(ref)			(ref)			(ref)			(ref)		
Yes	0.03	-0.27 to 0.34	0.828	-27.76	-51.73 to -3.79	**0.023**	-17.89	-31.85 to -3.93	**0.012**	-48.66	-89.33 to -7.98	**0.019**	-6.51	-11.85 to -1.17	**0.017**
**Endocrine comorbidities[6]**															
No	(ref)			(ref)			(ref)			(ref)			(ref)		
Yes	-0.69	-1.40 to 0.03	0.061	-71.51	-90.72 to -52.29	**<0.001**	-35.02	-48.68 to -21.36	**<0.001**	-116.28	-151.69 to -80.86	**<0.001**	-4.00	-15.42 to 7.42	0.493
**Nicotine use**															
No or former	(ref)			(ref)			(ref)			(ref)			(ref)		
Occasional or active	0.03	-0.12 to 0.17	0.716	-3.03	-14.48 to 8.43	0.604	-5.11	-11.26 to 1.03	0.103	-11.29	-31.12 to 8.54	0.265	2.19	0.30 to 4.08	**0.023**
**Alcohol consumption**															
Never	(ref)			(ref)			(ref)			(ref)			(ref)		
Sometimes or often	-0.02	-0.16 to 0.13	0.826	-2.82	-15.03 to 9.39	0.650	-0.09	-6.71 to 6.53	0.979	-3.73	-26.58 to 19.13	0.749	-0.38	-2.25 to 1.49	0.690
**Ejaculation abstinence[7] (days)**															
[0-2[	(ref)			(ref)			(ref)			(ref)			(ref)		
[2-7]	0.66	0.53 to 0.78	**<0.001**	32.39	22.78 to 41.99	**<0.001**	8.18	2.21 to 14.16	**0.007**	59.92	43.09 to 76.74	**<0.001**	-0.96	-2.72 to 0.80	0.284
>7	1.12	0.79 to 1.45	**<0.001**	112.57	69.76 to 155.38	**<0.001**	49.31	26.90 to 71.72	**<0.001**	267.87	160.99 to 374.75	**<0.001**	-6.35	-10.81 to -1.89	**0.005**
**BMI[8]**	-0.01	-0.03 to 0.01	0.235	-0.49	-2.07 to 1.10	0.548	-0.20	-1.07 to 0.67	0.654	-2.40	-5.20 to 0.41	0.094	0.29	0.06 to 0.53	**0.015**

Statistically significant p-values ($p < 0.05$) are shown in bold.

[1] Adjustment was made on age, cryptorchidism, endocrine comorbidities, nicotine use, alcohol consumption, days of abstinence and body mass index

[2] Total motile sperm count: volume × sperm concentration × total motility (a + b + c)

[3] All motile sperm (a + b + c); a = fast progressive movement, b = slow progressive movement, c = non-progressive movement

[4] Computed from linear regressions and the robust Huber-White (HC3) method

[5] Confidence interval

[6] Reported or documented prior to participating in the study (diabetes, thyroid disease, adrenal disease, Cushing syndrome, other)

[7] Abstinence period refers to the number of days between the last ejaculation and semen sample collection

[8] Body mass index
